# Peptide-targeted nanoparticles for tumor therapy

**DOI:** 10.1016/j.jconrel.2025.114195

**Published:** 2025-09-07

**Authors:** Valeria Sidorenko, Allan Tobi, Kazuki N. Sugahara, Tambet Teesalu

**Affiliations:** a Laboratory of Precision and Nanomedicine, Institute of Biomedicine and Translational Medicine, University of Tartu, Ravila 14b, 50411 Tartu, Estonia; b Division of GI/Endocrine Surgery, Department of Surgery, Columbia University Irving Medical Center, New York, NY 10032, USA; c Materials Research Laboratory, University of California, Santa Barbara, CA 93106, USA

**Keywords:** Tumor-homing peptides, Nanoparticle drug delivery, tumor microenvironment, Targeted therapy, Tumor-penetrating peptide, in vivo phage display

## Abstract

Most chemotherapeutics distribute non-specifically throughout the body, resulting in off-target toxicities. Nanoparticle (NP) formulations provide a strategy to improve drug delivery by extending circulation time, protecting therapeutic agents from degradation, and enabling controlled release. However, delivering NPs effectively to solid tumors remains challenging due to the barriers within the tumor microenvironment. Tumor-homing peptides address this challenge by targeting specific molecular markers accessible in tumors. These peptides bind to receptors on the tumor vasculature, facilitating drug delivery with reduced off-target effects. Unlike physicochemical approaches, which focus on optimizing NP properties such as size and charge but often lack specificity, tumor-homing peptides exploit molecular differences between healthy and malignant tissues to enable targeted delivery. A subclass of these peptides, known as tumor-penetrating peptides, promotes NP extravasation and tissue penetration, allowing delivery to cell populations deep within the tumor parenchyma.

This review examines the use of tumor-homing peptides for NP delivery, focusing on their role in improving tumor accumulation, overcoming microenvironmental barriers, and enhancing therapeutic outcomes.

## 1. Introduction

Chemotherapy remains a cornerstone of cancer treatment. Ideally, systemically administered anticancer drugs would selectively and effectively accumulate at tumor sites, minimizing toxicity to healthy tissues. However, most small-molecule chemotherapeutics lack cancer-specific targeting and distribute broadly in the body, leading to off-target effects and damage to normal tissues. Common adverse effects include immunosuppression, nausea, alopecia, fatigue, myelosuppression, neuropathy, mucositis, cardiotoxicity, hepatotoxicity, nephrotoxicity, dermatological issues, cognitive impairments, infertility, and an increased risk of secondary malignancies [1]. These adverse effects compromise patients’ quality of life and limit the therapeutic efficacy of chemotherapy, underscoring the need for more targeted and efficient drug delivery strategies.

Effectively targeting malignant cells and malignancy-supporting stromal cells within solid tumors requires overcoming the heterogeneous tumor microenvironment and its physical and physiological barriers. These include abnormal vasculature, dense extracellular matrices, hypoxic regions, and elevated interstitial fluid pressure, which hinder drug delivery and efficacy [2-5]. Increasing drug doses can enhance intratumoral concentrations, but this approach raises the risk of systemic toxicity and promotes multidrug resistance in cancer cells [6-9].

Nanoformulation of chemotherapeutics can extend drug circulation time by reducing renal clearance and protecting drugs from degradation [10,11]. Engineering nanoparticles (NPs) with programmable release properties enables controlled and sustained drug delivery [11-14]. Strategies to enhance NP accumulation at disease sites include physicochemical targeting and affinity ligand-based targeting. Physicochemical approaches include optimization of NP size, charge, surface chemistry, and hydrophobicity to act in concert with tissue microenvironmental properties like vascular permeability, pH, and redox potential [15-17]. However, these features are often shared by both healthy and diseased tissues, limiting specificity. Affinity targeting involves coating NPs with ligands, such as peptides, antibodies, or low-molecular-weight molecules like folic acid and bisphosphonates, to recognize tissue and disease-specific vascular signatures and enhance selective accumulation [18,19].

Recent advances in peptide-guided NP delivery are helping to overcome long-standing challenges in tumor targeting, where often less than 1% of administered NPs reach tumors. Optimized NP design—including tailored lipid compositions, surface functionalization, and precise control of peptide density and conjugation chemistry—improves stability, biodistribution, and cellular uptake. Homing peptide-guided targeting can dramatically enhance tissue accumulation, while strategies such as co-targeting multiple receptors, combining tumor-penetrating peptides, and leveraging AI-assisted peptide selection further increase delivery efficiency. Importantly, homing peptides target conserved binding sites, reducing species-specificity concerns, but clinical validation remains essential. These developments highlight the promise of peptide-guided NPs and underscore the relevance of systematically exploring their design, optimization, and translational potential in cancer therapy.

This review examines the principles and recent advancements in utilizing vascular signature-recognizing tumor-homing peptides for targeted NP delivery, with a focus on strategies to enhance NP distribution, overcome barriers, and improve therapeutic outcomes in solid tumors (Fig. 1).

## 2. Affinity targeting with homing peptides

### 2.1. Tissue and disease-specific molecular ZIP Codes

Blood and lymphatic vessels permeate all tissues (except cartilage, cornea and lens of the eye), serving critical roles in nutrient and waste exchange as well as complex physiological functions, including immune surveillance, blood pressure regulation, tissue homeostasis, intercellular communication, and tissue repair and regeneration. Endothelial cells that compose the inner lining of these vascular networks communicate with mural cells to develop and maintain the vascular system and participate in morphogenic and transport pathways.

Endothelial cells exhibit variability in shape, size, and structural organization, and this morphological diversity is essential for regulating blood flow, permeability, and responses to stimuli, contributing to the functional specialization of endothelial cells across different vascular regions and pathologies [20,21]. For example, endothelial cells in the blood-brain barrier form tight junctions and closely interact with surrounding mural cells, creating a highly selective and impermeable vascular barrier [22,23], whereas those in the liver sinusoids are fenestrated, lack a basement membrane, and exhibit a more irregular shape to facilitate the exchange of macromolecules and metabolites [24]. The cuboidal shape of lymphatic endothelial cells enhances fluid absorption, supports vessel remodeling and valve formation, facilitates immune cell trafficking through increased adhesion molecule expression, and provides structural integrity to withstand fluctuating pressures [25,26]. In solid tumors, blood vessels often exhibit abnormal morphological features, including irregular shape, disorganized architecture, enlarged lumen, and frequent tortuosity, which contribute to impaired blood flow and enhanced permeability [5,27]. Although it was known that endothelial cells vary between organs, the full appreciation of microvascular endothelial heterogeneity was supported by Auerbach’s 1981 discovery that endothelial cells express organ-specific antigens [28]. This discovery was later translated into the concept of “vascular ZIP codes” by Erkki Ruoslahti [29]. He deduced that the organ-specific molecular signatures on endothelial cells may direct cancer cells to achieve organ-specific metastasis and further speculated that the signatures can be used as a molecular target to systemically deliver drugs to selected sites. The *in vivo* peptide phage display technology (discussed below) has enabled unbiased discovery of peptides that can target the endothelial cell signatures. By identifying the binding partners (receptors) of these peptides, this approach has significantly advanced the mapping of vascular heterogeneity in health and disease. Ideally, a receptor for an affinity-targeting ligand should have target epitopes that are accessible via the administration route, display high selectivity, specificity, and surface density at the target site, avoid causing adverse effects upon engagement, enable ligand-receptor binding to achieve the desired therapeutic or diagnostic outcomes, and facilitate appropriate subcellular trafficking [30].

Tumor vasculature differs from normal tissue vasculature both morphologically and biochemically. A dominant feature of tumors is angiogenesis, as tumor growth requires new blood vessels to supply oxygen and nutrients [31]. Blood vessels undergoing angiogenesis, whether in tumors or regenerating normal tissue, express molecular markers not found on quiescent vessels. Tumor vessels, however, are morphologically distinct—they are typically tortuous, irregularly branched, and often deficient in pericytes, contributing to abnormal perfusion and leakiness. Biochemically, tumor vessels express a range of angiogenesis-related molecules, including integrins, endothelial growth factor receptors, proteases, cell surface proteoglycans, and ECM components [32]. Many of these molecules play active roles in angiogenesis and serve as targets for tumor-homing peptides. For example, as discussed below, RGD peptides recognize integrins on angiogenic endothelium, while NGR peptides bind aminopeptidase N expressed in tumor vessels of specific tumor types. Vascular heterogeneity is influenced not only by tumor type but also by the stage of tumor progression. Vascular heterogeneity is influenced not only by tumor type but also by the stage of tumor progression. Phage display profiling in a pancreatic islet carcinogenesis model identified peptides with distinct preferences for angiogenic progenitor lesions, fully developed tumors, or both, while showing no appreciable binding to normal tissues [33]. A similar stage-dependent pattern was observed in a transgenic squamous cell carcinoma model [34], underscoring the existence of stage-specific vascular “ZIP codes”. Beyond the vascular barrier composed of endothelial cells, targetable markers are expressed on tumor cells, cancer stem cells, various stromal cells in the tumor microenvironment, and the extracellular matrix (ECM) [35,36]. Whereas these parenchymal signatures are to some degree accessible due to inherent “leakiness” of tumor vasculature, their accessibility can be greatly improved by tumor penetrating peptides (discussed below).

Direct affinity targeting of malignant cells has proven a successful strategy for treating blood malignancies, with examples including CD19 and CD20 on B-cell malignancies [37,38], CD22 on acute lymphoblastic leukemia [39,40], CD33 on acute myeloid leukemia [41,42], and CD38 on multiple myeloma [43,44]. Similarly, malignant cells in solid tumors present on the surface overexpressed or mutated proteins, altered glycoproteins or glycolipids, or cancer-associated fetal proteins. Examples of such overexpressed surface receptors include HER2, CD19, CD20, CD133, and the endothelial growth factor receptor (EGFR) [45]. Another recurring theme is the surface expression of proteins in cancer cells that are typically localized in the cytosol, nucleus, or other intracellular compartments in healthy cells, such as nucleolin, p32, annexin A2, certain heat shock proteins, histones, and RNA binding proteins [46-51]. Cancer stem cells (CSCs), a subpopulation of cancer cells responsible for tumor initiation and relapse, possess high plasticity, making them particularly adept at acquiring resistance to treatments. Recent research has identified a range of potential molecular targets for CSCs, including CD24, CD44, EpCam, CLL-1, and others [52-56].

Immune cells, such as tumor-associated macrophages (TAMs) in the tumor microenvironment support essential functions of tumor progression, including angiogenesis, cancer cell proliferation, ECM remodeling, metastasis, immunosuppression, and resistance to therapy [57]. Thus, they provide an additional target for cancer therapies aimed at depleting protumoral TAMs or reprogramming these cells to adopt an antitumoral phenotype [58]. For example, the reprogramming of TAMs from the protumoral M2 phenotype to the antitumoral M1 phenotype can be achieved by targeting molecules such as CSF-1R or inhibiting the activity of MMP-9, which plays a role in ECM remodeling [59,60]. Molecular targets that are presented on protumoral M2 TAMs and can serve as receptors for affinity targeting ligands include CD68, CD163, CD206, MMP-9 and COX-2 [61].

Regulatory T cells (Tregs) are another set of immune cells that drive an immunosuppressive microenvironment in various solid tumors, leading to cancer progression and resistance to immunotherapy [62]. Tregs generally express cell surface molecules such as CD25, cytotoxic T-lymphocyte-associated protein 4 (CTLA-4), programmed cell death-1 (PD-1), and the glucocorticoid induced TNF receptor, which have been used to successfully target Tregs [63-66]. However, most of these molecules are not specific to tumor-infiltrating Tregs, and targeting them can cause systemic Treg depletion accompanied by serious autoimmune related toxicities [62,67,68]. Recently, several cell surface molecules, such as CCR8 [69-72] and αvβ5 integrin [73], have been shown to be preferentially expressed on tumor-infiltrating Tregs providing novel strategies to achieve tumor-specific Treg depletion.

Tumor-associated lymphatic vessels play a crucial role in tumor-host communication, metastatic dissemination, and the regulation of anticancer immune responses, including those to immunotherapy [74]. In adults, lymphatic vessels are mostly quiescent. However, when exposed to cytokines and growth factors released, for instance, by tumor cells or leukocytes, they become activated and can expand, enlarge, and undergo lymphangiogenesis [75]. Tumor-induced increase in lymphatic vessel density has been observed in a variety of human tumors [76]. Similar to blood vessel endothelial cells, lymphatic endothelial cells also present tumor antigens that could be used as receptors for affinity ligands, such as p32/gC1qR, VEGFR-3 and NRP-2 [48,77].

The ECM is a key tumor component, providing structural support, modulating the microenvironment, and serving as a source of signaling molecules. Desmoplastic tumors with extensive ECM include pancreatic ductal adenocarcinoma (with ECM contributing up to 60-80% of the tumor’s total mass), breast cancer (20-40%), hepatocellular carcinoma (20-50%), colon cancer (30-50%), and non-small cell lung cancer (20-30%), with ECM compositions rich in collagen, fibronectin, proteoglycans (e.g., syndecan, decorin), and glycosaminoglycans (e.g., hyaluronan, chondroitin sulfate), contributing to tumor stiffness, progression, and resistance to treatment [78,79]. Oncofetal ECM isoforms, linked to stromal activation, provide opportunities for affinity targeting. Tumor-associated ECM molecules are more abundant than cell surface receptors, making them less susceptible to target saturation—an important aspect in affinity targeting strategies [80]. In addition, unlike receptors overexpressed on malignant cells, ECM molecules are primarily deposited by genetically stable nonmalignant cells, offering more reliable targeting compared to molecules expressed by hypermutated malignant cells. Examples of ECM components utilized for cancer targeting include hyaluronan (HA), collagens, tenascin-C (TNC) and extra domain B splice variant of fibronectin (FN-EDB), versican, and biglycan [81,82].

Targeting extravascular cells and components within solid tumors often relies on the enhanced permeability and retention (EPR) effect mediated by the increased baseline vascular permeability in tumors [83]. However, passive targeting achieved through this effect can be unreliable due to various factors, such as the heterogeneity of vascular leakiness across the tumor and the challenge to overcome the high interstitial fluid pressure in many solid tumors [84]. Tumor penetrating peptides, discussed in the section 3.5 may help overcome these obstacles and improve delivery to extravascular targets.

### 2.2. Mapping of vascular heterogeneity by phage display

Target-engaging peptides are identified through various rational and agnostic approaches including the adoption of interaction modules from naturally occurring proteins, diverse *de novo* design approaches, and screening of combinatorial peptide libraries [85,86]. Phage display, first developed by George Smith in the 1980s, utilizes genetically engineered bacteriophages to present randomized peptides or polypeptides on their surface as genetic fusions to coat proteins [87]. This technique enables unbiased identification of ligands for specific molecular targets. Phage biopanning can be performed on targets of different complexity, ranging from purified molecules, to cells, and to complex *in vivo* targets (Fig. 2). A major breakthrough was reported in 1996 when Erkki Ruoslahti’s laboratory pioneered *in vivo* phage display as an approach to discover tissue- and disease-specific vascular homing peptides [88]. In this technology, peptide phage libraries are administered to live animals, followed by rescue of phages from the target site, and their amplification and use for another round of biopanning. Through repeated phage selection rounds, peptides that confer tropism to specific organs or disease sites are identified. These peptides are then used for targeted delivery of payloads and for the identification of their binding partners or receptors [89-92]. *In vivo* biopanning combines positive selection at the target site with negative selection in nontarget tissues, thereby reducing the selection of promiscuous binding peptides and minimizing background noise. An important consideration is that T7 phages, commonly used for *in vivo* biopanning, are themselves biological NPs. The size (~55 nm) and aspect ratio of the T7 nucleocapsid are more similar to clinically used nanoparticles than filamentous phages, and the lytic T7 system is less restrictive in the sequence space of displayed peptides [93]. In the T7-Select system (Novagen), peptides are displayed C-terminally on the major coat protein 10A at ≤ 415 peptides per phage particle. T7 phages can be used to distinguish internalized from non-internalized peptides. Following binding, exposed phages can be selectively inactivated using an acidic wash or perfusion, while internalized phages remain protected, allowing identification of peptides that are actively taken up by cells [94]. The phage libraries used for in vivo display consist of collections of clones presenting genetically encoded randomized peptides fused to this C-terminus [95], typically covering ~10^9^ different sequences, approaching the theoretical diversity of a seven-amino-acid random library (1.28 × 10^9^). *In vivo* phage biopanning of naïve libraries can be viewed as a first line discovery tool that yields homing peptides for proof-of-concept targeting studies and for receptor hunt. The receptor can then be used to develop optimized second-generation homing peptides or alternative affinity ligands, such as antibodies or small molecules. This concept is illustrated by studies on p32-targeting peptides from the Ruoslahti laboratory. First, Laakkonen and coworkers used *in vivo* phage display to discover LyP-1 peptide with tropism to breast tumor lymphatics and malignant cells [96]. Subsequently, Fogal and coworkers used affinity chromatography to identify p32 as a LyP-1 receptor expressed on the lymphatic endothelial cells, malignant cells, and activated macrophages [97]. Finally, Paasonen and coworkers used cell free biopanning to identify a second generation p32 binding peptide, linTT1, and screened for low molecular weight compound libraries to identify p32 binding small molecule ligands [98].

In recent years, numerous variations and technological advancements have enhanced the *in vivo* biopanning technique. In particular, high-throughput sequencing of phage libraries has enabled comprehensive monitoring of the peptide landscape in target and control organs throughout the screensacilitating the selection of the most prevalent candidates within the target tissue [86,99]. Additionally, variations of phage biopanning, including *in vitro* screening and exposure to external actuators like proteases, can be employed to identify peptides with certain desired properties. For example, tumor homing peptides PL1 and PL3 that selectively bind to two separate target receptors were identified using cell-free phage display on immobilized tenascin-C and fibronectin [100,101]. In another example, phages were used to select for peptides that bind to the target recombinant protein only after being specifically cleaved by a tumor-derived protease [102]. Either live or fixed cells can be used for the *in vitro* phage display [103]. Similarly, *ex vivo* phage display has been also used to identify targeting peptides, although to a lesser extent [104]. Cell-based and organoid screening platforms enable selection against physiologically relevant cell types to capture complex cell-surface epitopes and microenvironmental cues.

Whereas the current review primarily focuses on peptides developed through phage display, it is important to note that several alternative technologies exist for designing or to aid development of target-specific homing peptides. These include in vitro display techniques such as mRNA and ribosome display, computational and structure-based design, and synthetic peptide libraries including one-bead-one-compound (OBOC) approaches [105]. Together, these methods broaden the peptide discovery toolbox and complement phage-based strategies.

Panel A illustrates the *in vitro* phage biopanning process, where a library of phages displaying randomized peptides is exposed to a target of interest, such as a protein or cell surface receptor. After incubation, non-binding phages are washed away, and the bound phages are eluted and amplified for subsequent rounds of selection. Panel B depicts the *in vivo* phage biopanning process, which involves injecting the phage library into a living organism, such as a mouse, to allow the phages to interact with biological targets within their natural microenvironment. After several rounds of selection, the phages that bind to the target tissue or receptor are isolated, providing peptides that are more likely to be effective in therapeutic or diagnostic applications due to their biological relevance. High-throughput sequencing of peptide-encoding region of phage genome and bioinformatics analysis is used to analyze the peptide landscape through the screens in both cases [107].

### 2.3. Precision NP delivery: the role of tumor-homing peptides

The clinical success of NPs is evident from the approval of more than 15 cancer nanomedicines [108]. Furthermore, the widespread use of lipid NP (LNP)-based mRNA vaccines during COVID-19 pandemic has further highlighted the medical potential of nanotechnology [109]. Currently approved cancer nanotherapeutics rely on passive targeting due to the EPR effect that results from leaky tumor blood vessels and deficient lymphatics [83]. Clinical experience with Doxil, a PEGylated liposomal formulation of doxorubicin [110], highlights the challenges faced by the current generation of nanodrugs. While the liposomal formulation enhances biodistribution and pharmacokinetics—reducing systemic toxicity and increasing tumor accumulation through prolonged circulation—it has not significantly improved overall survival compared to free doxorubicin therapy [111,112]. Despite promising developments, affinity-targeted nanomedicines have yet to pass clinical testing and gain FDA approval (@@Rosenblum et al. 2018). For example, BIND-014, an affinity-targeted nanoformulation of docetaxel, failed in Phase II clinical trials due to a lack of significant therapeutic advantage over standard docetaxel [113]. A potential cause of the failure was the variable expression level of its target, prostate-specific membrane antigen (PSMA), among individuals and tumor subtypes.

Over the past two decades, researchers have developed a diverse array of tumor-homing peptides to enhance the specificity and efficacy of anticancer NP systems [114-116]. As affinity targeting ligands, homing peptide have several favorable translationally relevant features, such as lack of species specificity, low immunogenicity and cost-effective production (Fig. 3). Whereas homing peptides generally have low affinity (in the low micromolar or high nanomolar range), conjugating peptides to NPs enables their multivalent presentation on NP surfaces, increasing their binding strength to target tissues and significantly improving both specificity and therapeutic efficacy [117]. Broadly, tumor-targeting peptides can be categorized into vascular docking peptides, which remain confined to the vascular endothelial barrier, and tumor-penetrating peptides, which—after initial recruitment—activate biological pathways that promote extravasation and deep penetration into the tumor parenchyma (Fig. 4). While this classification is not absolute, it provides a useful framework for understanding peptide mechanisms and applications.

Vascular homing peptides possess features that make them particularly well-suited for targeted delivery applications. These include moderate affinity, which allows to bypass the "binding-site barrier" that limits the penetration of high-affinity molecules like antibodies into solid tumors [118,119]. Additional advantages of peptides include their smaller size, which enables straightforward synthesis, eliminates the need for complex antibody engineering, and provides cost efficiency. Peptides exhibit low immunogenicity, high biocompatibility, and scalability, and can be engineered with non-natural modifications to increase stability and for site-specific conjugation, enhancing stability and target binding [120,121]. Importantly, as short peptides tend to interact with conserved and functionally important binding pockets on the surface of target molecules, they often possess biological activity across species. As postulated by Tim Hunt in 1990, biological systems frequently depend for recognition and targeting activities on short linear peptide motifs (SLiMs) consisting of 3 to 11 contiguous amino acids [122,123]. As the smallest structural and functional components, SLiMs undergo convergent evolution with minimal mutations, and their low affinity promotes transient interactions critical for dynamic processes like cell signaling and adhesion.

The following sections review key tumor-homing peptides, the types of NPs and theranostic cargos they deliver, and the corresponding tumor models in which they have been evaluated (Table 1).

### 2.4. Homing peptides for delivery of NPs to the tumor vasculature

Vascular heterogeneity is most simply targeted by vascular homing peptides that engage with receptors on the surface of tumor vessels without triggering pathways that lead to secondary interactions or spreading. First set of such peptides interacts with cellular adhesion receptors overexpressed on tumor vessels, integrins [124]. This family of 24 transmembrane heterodimeric receptors, composed of various combinations of α and β subunits, is expressed across diverse cell types and tissues. Integrins play crucial roles in numerous cellular processes, including adhesion, migration, signaling, motility, proliferation, polarity, differentiation, and survival [125]. Among the integrin subtypes, αvβ3 and αvβ5 are particularly overexpressed in angiogenic and activated blood vessels, including those within tumors [126]. Notably, integrins recognize their target ligands through simple peptide motifs, such as the RGD (arginine-glycine-aspartic acid) tripeptide, originally identified as the core recognition sequence for fibronectin-binding integrins [127]. Subsequent studies revealed the presence of the RGD motif in other ECM proteins, leading to the development of numerous peptidic and non-peptidic integrin ligands based on this sequence, each with varying specificity [128]. A well-studied example is cilengitide, a cyclic RGD-mimetic peptide (sequence: RGDfV) that specifically targets αvβ3 and αvβ5 integrins [124]. Initially developed as an antiangiogenic agent [129], cilengitide was later found to have an intrinsic antitumor activity [130]. The CENTRIC trial tested cilengitide in combination with standard radiotherapy and temozolomide for newly diagnosed glioblastoma (GBM) patients with methylated MGMT promoters [131]. Despite initial promise in preclinical studies, the trial found that cilengitide did not significantly improve overall survival or progression-free survival compared to standard therapy. Although well-tolerated, cilengitide’s failure to enhance treatment efficacy led to the discontinuation of its development for GBM [132]. Beyond cilengitide, other RGD-containing peptides have been developed for tumor targeting and drug delivery applications. CDCRGDCFC (referred to as RGD-4C), a cysteine-rich cyclic RGD peptide that selectively binds αv integrins [133,134]. RGD-4C benefits from enhanced stability and binding affinity due to its constrained structure, and achieves tumor-specific delivery of proteins and NPs. For example, RGD-4C has been fused to saponin, a plant-derived single chain ribosome inactivating protein [135], and to an anti-TNFα nanobody [136] to target and reduce the growth of tumors in mice. RGD-4C has also successfully delivered doxorubicin-loaded apoferritin nanocages to subcutaneous tumors of U87MG human glioblastoma in mice in an αvβ3 integrin-dependent manner, leading to improved tumor uptake and tumor-specific cytotoxicity compared to doxorubicin alone [137]. The CRGDC peptide, another cyclic RGD derivative [138], has also been employed for conjugation with NPs, such as ironoxide nanoworms, to enhance tumor-specific accumulation [139,140].

Another molecule expressed on tumor endothelial cells is aminopeptidase N (APN or CD13) that serves as the receptor for the NGR peptide (sequence: CNGRC) [141]. The NGR peptide was identified through screening of random peptide libraries displayed on phage for their ability to home to human breast tumor xenografts *in vivo* [142]. NGR is effectively a multitarget peptide, as in the bloodstream, NGR peptide is deamidated, and the resulting isoDGR peptide acquires the ability to bind to integrins [143]. This illustrates an important role of secondary interactions of the homing peptides triggered in the plasma or in the tumor microenvironment. Whereas the clinical development efforts on NGR are focused towards delivery of protein payloads such as tumor necrosis factor-alpha [144], various NGR-functionalized nanoplatforms have been explored to enhance tumor targeting and therapeutic efficacy. For example, NGR-targeted liposomal doxorubicin (NGR-DOX) demonstrated improved tumor accumulation, reduced systemic toxicity, and enhanced therapeutic outcomes in neuroblastoma and fibrosarcoma models [145]. Similarly, NGR-modified PEG-PLGA NPs exhibited prolonged circulation, controlled drug release, and increased tumor specificity, leading to improved efficacy in xenograft models [146]. Beyond drug delivery, NGR-functionalized gold NPs have been utilized for targeted photothermal therapy, while NGR-SPIONs have shown potential in MRI imaging by enhancing contrast in CD13-positive tumors, improving both diagnosis and treatment monitoring [146].

Some vascular homing peptides target proteins that are exposed on the cell surface in the tumor vessels while they remain intracellular under normal conditions. For example, F3 peptide targets nucleolin – a nucleolar protein misplaced at the cell surface on activated endothelial cells in tumor vasculature, as demonstrated by Christian et al. [147]. The nucleolin-targeting 31-mer peptide F3 was originally identified through *in vitro* phage screening on mouse bone marrow cells and demonstrated *in vivo* targeting capability by homing to HL-60 human leukemia xenografts [148]. In a study by Qin et al., F3-conjugated hydrogel NPs improved uptake by glioma (9L) and breast cancer (MCF-7) cells, with higher uptake in glioma cells, and efficiently delivered doxorubicin [149]. In another study, F3 peptide-functionalized liposomes were found to selectively targeted nucleolin-overexpressing breast cancer stem-like and non-stem cells in triple negative breast cancer, linking nucleolin expression to tumorigenicity and enabling complete cell death through synergistic drug delivery [150].

In the examples above, homing peptides primarily facilitate the delivery of therapeutic payloads to tumor-associated blood vessels. However, achieving a therapeutic effect requires overcoming multiple biological barriers to ensure that the payloads reach malignant cells and tumor-supporting stromal cells (Fig. 5). The first barrier is the tumor vasculature itself, which often exhibits irregular vessel architecture and compromised perfusion to limit drug penetration. Once extravasated, NPs encounter the dense ECM and high interstitial pressure, which act as physical and biochemical barriers to diffusion, restricting the passive movement of therapeutics and further hindering their deep penetration. Finally, cellular uptake of therapeutics is another challenge. Many tumor cells and associated stromal cells have mechanisms to resist drug accumulation, including efflux transporters and endosomal sequestration, which limit intracellular bioavailability. To address these challenges, targeting peptides with ability to traverse tumor barriers must be employed.

### 2.5. Overcoming barriers: peptide-driven NP delivery to the tumor parenchyma

Following docking to the tumor vessels, some homing peptides activate biological pathways that promote extravasation and deep penetration into the tumor parenchyma. A subclass of such peptides, termed tumor penetrating peptides (TPP), are defined by the presence of an R/KXXR/K motif, which mediates neuropilin-1 (NRP-1)-dependent extravasation and a subsequent tumor penetration cascade [151]. The prototypic TPP, iRGD, was discovered through *ex vivo* and *in vivo* phage display screening in mice with experimental bone metastases from human prostate cancer cells [91]. Across three independent screenings, peptides with the CRGD(K/R)GP(D/E)C sequence were consistently enriched, suggesting a strong targeting preference. CRGDK/RGPD/EC peptides effectively bound to and internalized into tumor cells, leading to the designation of the peptide internalizing RGD or iRGD in short. The iRGD sequence features a basic residue following the RGD motif, and the RGD(K/R) motifs [and more broadly, (R/K)XX(R/K) containing peptides] turned out to constitute a conditional NRP-1 binding motif that can be switched on by proteolytic cleavage to C-terminally expose the motif [152]. This requirement for C-terminal exposure prompted us to call the phenomenon C-end Rule (CendR, pronounced “sender”). Mechanistic studies showed that iRGD acts in three steps: binding to integrins, proteolytic processing to expose a CendR motif, and subsequent NRP-1 binding to activate the CendR extravasation and tissue penetration pathway (Fig. 6). This process facilitates tumor penetration by enhancing peptide transport across blood vessels and into tumor tissue via transcytosis. The pathway enables the delivery of various therapeutic molecules, including antibodies and NPs, into extravascular tumor tissue. iRGD is especially effective in penetrating fibrotic tumors such as pancreatic ductal adenocarcinoma (PDAC) by utilizing the tumor stroma as a conduit. The peptide’s ability to target integrin-expressing carcinoma-associated fibroblasts (CAFs) and immune cells, enhances its therapeutic potential in aggressive cancers [153]. iRGD has been widely used for NP targeting to improve drug delivery into tumors by enhancing penetration beyond the blood vessels. Numerous studies [151] have demonstrated the utility of iRGD conjugation to NPs to increase their accumulation and deep tissue penetration to potentiate the activity of therapeutic payloads, including chemotherapeutics and gene delivery systems. This strategy has shown particular promise in treating desmoplastic tumors like PDAC, where conventional drug delivery is hindered by the dense stroma and high interstitial pressure. Importantly, iRGD can be coadministered with non-targeted NPs rather than being directly conjugated to them [154] (Fig. 6). In this approach, iRGD binds to tumor vasculature, activates the CendR pathway, and enhances the permeability of the tumor microenvironment, allowing NPs to extravasate and penetrate deeper into tumor tissue. This peptide-based targeting of bystander nanoparticles for tumor delivery exploits the ability of iRGD to hijack the NRP-1-mediated vesicular transport pathway, which is similar to the vesiculo-vacuolar organelles (VVOs) induced by VEGF but with a crucial difference—iRGD requires proteolytic activation to initiate transport, ensuring tumor specificity [155,156]. Once activated, iRGD facilitates transcytotic vesicle formation, enabling the uptake of surrounding extracellular fluid and non-specifically transporting bystander molecules, including NPs and drugs, across the tumor vasculature. This allows for tumor-targeted drug delivery simply by coinjecting iRGD with therapeutic agents, bypassing the need for chemical modification of drugs or complex NP formulations. Importantly, coadministration of peptides instead of covalent conjugation can potentially limit recognition and uptake of decorated nanoparticles by macrophages, reducing clearance by the mononuclear phagocyte system and improving circulation time, which can enhance tumor accumulation and therapeutic efficacy. When peptides or other targeting moieties are covalently attached to the NP surface, they can alter surface charge, hydrophilicity, and protein corona formation, potentially increasing recognition and uptake by the mononuclear phagocyte system, including through scavenger receptor-mediated pathways [157-159]. In contrast, co-administration of free targeting peptides, such as iRGD, activates tumor-specific transport pathways without modifying the NP surface while enhancing their accumulation in tumors [154,160,161], thereby reducing opsonization and macrophage-mediated clearance. Another advantage of this approach is that multiple drugs can be delivered simultaneously without requiring affinity ligand conjugation, which often complicates formulation and regulatory approval. Moreover, since iRGD activates a transport pathway that accommodates any molecule fitting into the vesicles, drug delivery is not limited by receptor saturation and continues as long as the pathway remains active. Similarly, a number of other CendR peptides have been shown to be effective in inducing transport of molecular and nanoscale bystander cargo across the vasculature and into the tumor tissue [162]. For example, co-administration of a tumor-penetrating CendR peptide (tLyp-1) was reported to enhance the delivery of F3-functionalized NPs, leading to increased tumor accumulation and prolonged survival in glioma-bearing mice [163]. iRGD is currently in multiple clinical trials as a co-injected enhancer of standard-of-care cancer therapies. The phase 1 CEND trial performed in stage 4 PDAC patients showed preliminary efficacy data that iRGD may double the response rate of co-injected gemcitabine and nab-paclitaxel without enhancing off-target cytotoxicity [164]. A follow up randomized phase 2 ASCEND trial in stage 4 PDAC patients (NCT0504212).

Inspired by iRGD, and by implementing rational design, the iNGR peptide (sequence: CRNGRGPDC) was developed as an enhanced version of the tumor targeting NGR peptide [166]. Similar to NGR, iNGR binds to aminopeptidase N expressed in the tumor vasculature, but it was engineered to incorporate a cryptic CendR motif to enable interaction with NRP-1 after proteolytic activation. This additional interaction triggers the CendR pathway, promoting tumor penetration and increasing the efficiency of drug and NP delivery. Braun *et al*. demonstrated that a prototypic CendR peptide with an exposed CendR motif (RPARSGR) can be *de novo* modified to produce a urokinase-activated variant RPARSGR↓SAGGSVA [167]. The cryptic variant showed preferential accumulation in urokinase-overexpressing tumor models and NRP-1-dependent internalization after proteolytic processing. As a further advancement, a modified cell-free phage display method was used to screen for urokinase-conditional cryptic variants of another known tumor targeting peptide with an exposed CendR motif, PL3 [168]. The study revealed novel urokinase-sensitive variants that retained tumor targeting properties comparable to the parent PL3 peptide while reducing off-site accumulation in healthy tissues, a known limitation of non-cryptic CendR peptides.

In addition to targeting endothelial cells of the vasculature and cancer cells, other direct and indirect approaches for targeting solid tumors beyond vascular barrier have been investigated. The tumor microenvironment (TME) offers a multitude of targets, such as immune cells, ECM components and CAFs, that may provide more stable and high-capacity targets compared to cancer for drug delivery and treatment options [169,170]. Targeting the unique composition of the tumoral ECM offers a promising alternative to conventional cell-targeting strategies. Upon binding, drug carriers can form localized depots within the ECM, from which the released chemotherapeutics diffuse to reach heterogeneous tumor cell populations, tumor-supportive cells, and tumor stem cells [171]. Recent advances in ECM profiling technologies have made it possible to map ECM signatures in patient tumors [172,173]. Approaches such as phage display using ECM-enriched preparations and LC-MS/MS-based proteomics have identified such signatures in triple-negative breast cancer and colorectal cancer, providing a basis for developing broadly applicable imaging and therapeutic targeting strategies. Among stromal cells, TAMs, especially protumoral M2-like TAMs as opposed to antitumoral M1-like TAMs, have garnered significant attention as they play a crucial role in the regulation of the tumor immune environment [174]. Therapeutic strategies are mainly aimed at either repolarizing M2 TAMs or eliminating them, both of which require specific targeting. For this purpose, several peptides have been identified. A mannose receptor CD206-binding peptide named UNO (sequence: CSPGAKVRC, cyclic) was identified through phage display conducted on mice with metastatic breast cancer [90]. UNO demonstrated homing and internalization in M2 TAMs across various solid tumor models [90]. Further analysis determined the minimal binding motif of UNO to CD206 as CSPGAK (referred to as mUNO), which notably contains a sequence common to all CD206-binding collagens [175]. The mUNO peptide also exhibited selective homing to M2 TAMs. Another CD206-targeting peptide RP-182 (sequence: KFRKAFKRFF) is a synthetic peptide identified through biophysical homology screening of host defense peptides, leveraging the Molly font tool to detect conserved structural motifs [176]. It induces a conformational shift that reprograms TAMs from a tumor-promoting M2-like phenotype to an antitumor M1-like state [176]. RP-182 enhances both adaptive and innate immune responses, showing efficacy in murine and patient-derived cancer models. Notably, it demonstrated potential to improve responses to chemotherapy and checkpoint blockade therapies, highlighting its promise in cancer immunotherapy.

### 2.6. Multitarget peptides for enhanced tumor delivery of NPs

Peptides that simultaneously target multiple molecules can utilize an increased number of available receptors to overcome a significant bottleneck in affinity targeting and precision delivery [177]. Furthermore, solid tumors are known to be highly heterogeneous, both intra- and inter-tumorally [178], and multitarget peptides may therefore provide a more uniform biodistribution of payloads by not having to rely on a single molecule.

Targeting the tumor microenvironment has been suggested as a more stable and reliable approach than targeting tumor cells, which can downregulate or lose specific antigens over time [179]. Oncofetal splice variants of components of ECM, such as FN-EDB and C-isoform of Tenascin-C (TNC-C), show robust upregulation in solid tumors compared to low baseline expression levels in normal adult tissues [178,180]. L19 antibody against FN-EDB has been developed into precision contrast agents and immunocytokines such as L19-IL2 and L19-TNFα, undergoing multiple clinical trials, including phase 2 studies, with promising interim results [181]. Neri et al. developed TN11, a high-affinity recombinant antibody fragment from a phage display library that specifically targeted the spliced repeat C of tenascin-C, identifying its selective expression in high-grade astrocytomas and its potential as a therapeutic agent [182]. A study inspired by this finding used peptide-phage biopanning to identify a dual-binding tumor targeting peptide PL1 (sequence: PPRRGLIKLKTS) to target recombinant human FN-EDB and TNC-C isoforms [101]. The PL1 peptide selectively binds both proteins, with critical binding interactions facilitated by the L6 and T11 amino acids. Molecular modelling studies revealed that the engagement of PL1 with both FN-EDB and TNC-C are mainly driven by electrostatic interactions, and interestingly, PL1 could induce internalization of conjugated NPs into cancer cells through a micropinocytosis-like endocytosis pathway [183]. PL1-functionalized NPs also showed increased accumulation in experimental tumor models, highlighting the peptide’s potential for facilitating targeted *in vivo* delivery of imaging agents and therapeutic agents [101].

Another example of a bispecific peptide is PL3 (sequence: AGRGRLVR), which was discovered using peptide-phage biopanning on recombinant TNC-C and isolating candidates based on their high affinity to both TNC-C and NRP-1 [100]. The C-terminal motif RLVR (CendR motif) is responsible for the binding of NRP-1, whereas the exact mode of engagement with TNC-C remains speculative, possibly driven by charge-based electrostatic interactions. ECM-targeting through TNC-C provides ample targets, whereas the NRP-1-dependent activation of the CendR pathway unlocks deep access into the tumor parenchyma, making PL3 a promising candidate for anticancer drug delivery. This is further supported by experiments with tumor-bearing mice that demonstrated efficacy of PL3 in improving delivery of various NPs as well as proapoptotic cargo, increasing the survival of mice [100].

In addition to screening for bispecific peptides, a feasible approach is to combine effective monospecific peptides to synthesize a dual-binding peptide. In this case, it must be ensured that the bispecific design does not interfere with the binding functionality of the combined peptide fragments. Ideally, the combined peptides should be synergistic or at least directed at separate targets. Kang et al. fused FHK (sequence: FHKHKSPALSPV) and tLyp1 (sequence: CGNKRTR) peptides through a cysteine residue to produce a bispecific peptide named Ft, which targets TNC-C and NRP-1, respectively [184]. They demonstrated that the activities of both original peptides have been preserved. Kang *et al*. speculated that the tLyp1 fragment would help cross the blood-tumor-barrier and ensure deep tumor tissue penetration via the CendR pathway while the FHK fragment promoted delivery to the neovascularized areas of the tumor. Indeed, the Ft peptide along with NPs was internalized into GBM cells, co-localized with TNC-C-rich areas, and provided a higher level of extravasation and penetration. Finally, the Ft peptide synergistically improved the survival of GBM-bearing mice when combined with drug-loaded NPs, validating the concept of dual-targeting.

## 3. Path forward: Overcoming challenges in peptide mediated tumor targeting

Despite preclinical and clinical advancements reviewed above, NP-based tumor targeting remains a major challenge. In many cases <1% of administered NPs reaches tumors [185], primarily due to biological factors such as immune clearance and protein corona formation, which significantly limit NP accumulation at the target site [186]. Some of these limitations can be mitigated through systematic optimization of NP physicochemical properties [186]. For instance, LNPs can be fine-tuned using combinatorial lipid libraries and modified phospholipid compositions to enhance stability and biodistribution [187,188]. This is particularly important with the emerging interest to achieve gene delivery to cancer cells and immune cells in the tumor tissue, which requires highly sophisticated NP delivery platforms to achieve subcellular targeting in a particular subset of cells within the tumor tissue.

### 3.1. Limited benefits of ligand-guided targeting

Actively targeted NPs also face criticism for limited benefits of ligand-guided targeting and overestimating targeting efficiency due to insufficient quantitative analysis and lack of rigor in study design. However, various reports have demonstrated the profound influence of active targeting on NP biodistribution and organ tropism. For instance, we routinely achieve with biological bacteriophage NPs displaying homing peptides up to two orders of magnitude greater accumulation at target sites compared to non-targeted phages. The key challenge remains replicating these high targeting efficiencies with synthetic NPs. Optimizing critical factors such as peptide density, linker design, and conjugation chemistry may provide important clues to overcome the challenges.

### 3.2. Need for systematic optimization of NP functionalization

We recommend that any new nanoplatform undergo a stage where well-characterized homing peptides are used to optimize NP target-ability. In our case, we prefer to use the prototypic CendR peptide RPARPAR, known to engage the cell surface receptor NRP-1 [152], in optimization studies. We first evaluate the effect of peptide density, linker design, and conjugation chemistry on NP-binding to immobilized receptors in cell-free binding assays. Next, cellular binding and internalization assays on NRP-1-positive cells are performed to assess uptake efficiency. Finally, since the RPARPAR peptide shows strong lung tropism, *in vivo* lung homing assays will confirm targeting efficacy in a physiological context and allow defining a concentration window for effective targeting. Once optimal functionalization parameters are established using a control peptide/receptor pair, these conditions can be applied to other homing peptides within the same nanosystem.

### 3.3. Restricted receptor availability and density

Even if active targeting is optimized, the delivery capacity of the system—dictated by the availability of homing peptide receptors on target cells or tissues—may still be insufficient to achieve the delivery of NPs at therapeutic concentrations. Hussain and Ruoslahti (2013) discuss this limitation in their work on peptide targeting, emphasizing that the concentration and local density of target receptors on the cell surface is a critical factor influencing the efficiency of NP delivery. In many cases, the target receptor may be present in limited quantities, either due to its biological expression level or because of the dynamic nature of receptor availability, which can vary depending on the tissue or disease state.

### 3.4. Strategies to enhance receptor availability and delivery capacity

It is important to explore alternative methods to achieve greater receptor availability. For example, using peptide combinations or co-targeting strategies, where multiple receptors or complementary pathways are targeted simultaneously (e.g. as in the case of bi- and multispecific peptides such as PL1, PL3, and SKLG), may increase the overall delivery capacity [168,189]. Additionally, optimizing the pharmacokinetics and biodistribution of the NPs—ensuring that they remain in circulation long enough to accumulate at target sites—can further enhance their therapeutic potential. Finally, coadministration of tumor penetrating peptides such as iRGD can enhance the extravasation of peptide NPs and enhance the number of systemically accessible receptors in tumor site [162]. Thus, while peptide-guided targeting holds significant promise for improving the specificity and efficiency of NP delivery, achieving therapeutic concentrations may still require strategies that address the availability and density of the target receptors, in addition to the optimization of targeting parameters.

### 3.5. Translational barriers and species specificity

Translating the affinity targeted nanosystems from animal studies to clinical applications must deal with the potential species specificity of the targeting ligand and differences in its receptor’s expression between the models and patients. Different from surface-embracing targeting ligands, such as antibodies and aptamers, homing peptides typically target conserved binding pockets on target proteins and are thus not species specific. However, the expression of accessible receptors of homing peptides must be carefully validated using clinical tumors or tissues collected during autopsies. Parallel auditioning of peptides using phage playoff assay on clinical samples, along with the integration of high-throughput sequencing-derived peptide representation data from individual and combined screens [107], can further accelerate the identification of homing peptides. Our laboratory routinely employs in vivo peptide phage display assays of phage pools presenting candidate homing peptides for comprehensive, internally controlled screening of organ- and tissue-specific delivery [107]. The same strategy can be applied to synthetic lipid nanoparticles or other nanocarriers, each barcoded with nucleic acids and functionalized with distinct homing peptides, to evaluate their suitability for specific delivery platforms. This approach, currently primarily used to optimize the physicochemical properties of nanoparticles [190], also enables systematic mapping and quantitative biodistribution analyses, including specific cell populations within tumors, facilitating the selection of optimal homing peptide–nanoparticle combinations. Machine learning and artificial intelligence (AI) are expected to play a central role in optimizing this process, as demonstrated by recent studies [191,192]. The application of these technologies can help decipher complex datasets, thereby enhancing the efficiency and accuracy of homing peptide discovery. Integrating such strategies with AI-driven analysis could further refine peptide selection, improving both the efficiency and precision of targeted delivery systems.

## 4. Summary and outlook

While significant challenges remain in NP-based tumor targeting, ongoing advancements suggest a path forward. By systematically refining key factors such as peptide density, conjugation chemistry, and receptor engagement, it is possible to improve targeting precision and increase NP accumulation at tumor sites. However, the success of these strategies will also depend on addressing biological constraints, including receptor availability and variability across different tissues and disease states. The integration of AI-enhanced peptide discovery, co-targeting strategies, and tumor-penetrating peptides may help overcome these barriers and enhance therapeutic delivery. As these approaches progress toward clinical translation, careful validation of clinical samples will be essential to ensure their effectiveness and translatability, ultimately bringing targeted nanomedicine to real-world applications in cancer treatment.

## Supplementary Material

Supplementary Materials file

## Figures and Tables

**Fig. 1.**
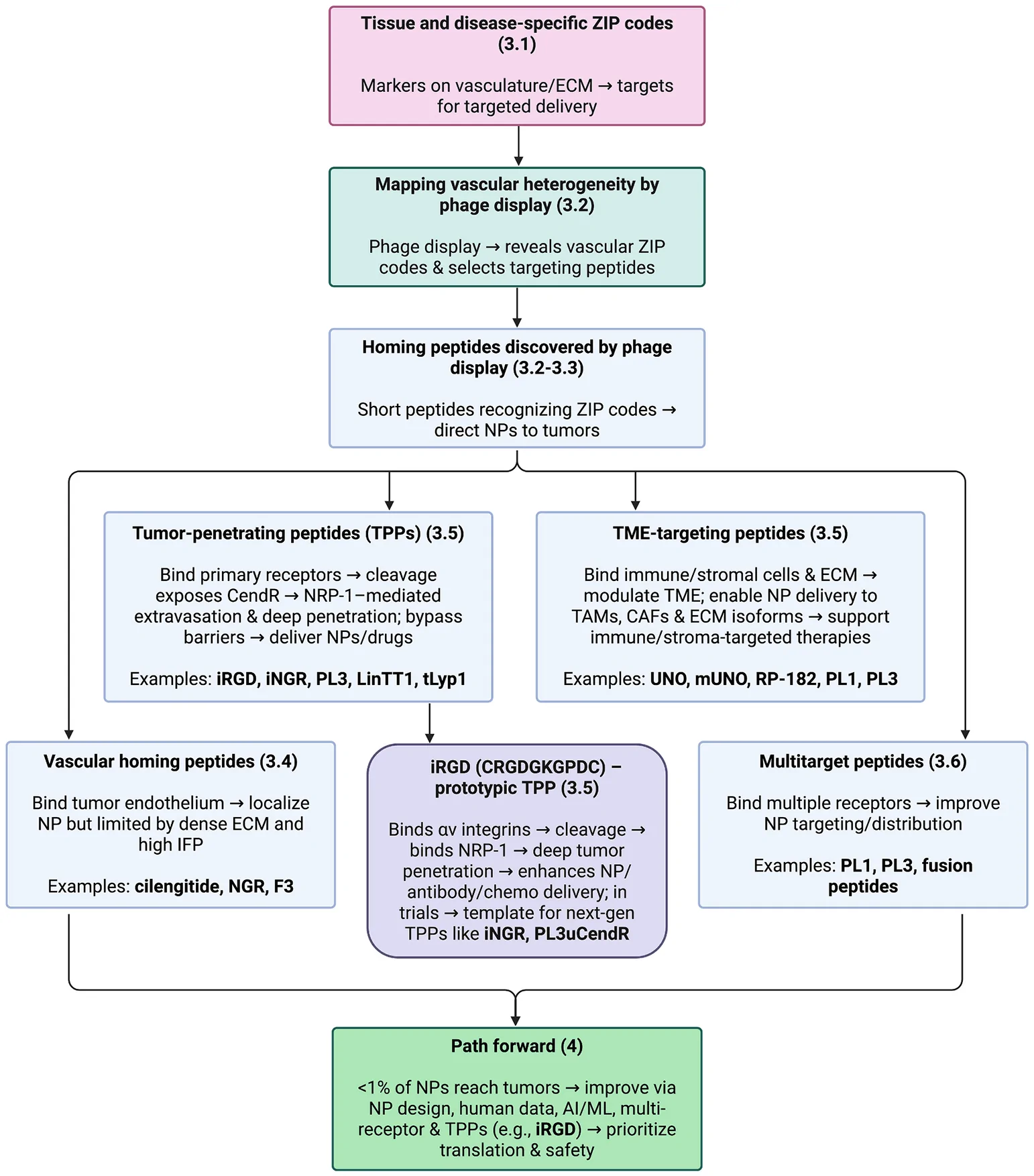
Overview of peptide-guided nanoparticle delivery to tumors. Tissue- and disease-specific vascular “ZIP codes” identified by phage display enable the discovery of homing peptides, grouped into vascular homing, tumor-penetrating, multitargeting, and tumor microenvironment (TME)-targeting classes, with iRGD highlighted as a clinically advanced tumor-penetrating peptide. The figure concludes with current challenges and future strategies for improving nanoparticle delivery and clinical translation. Created in BioRender.

**Fig. 2.**
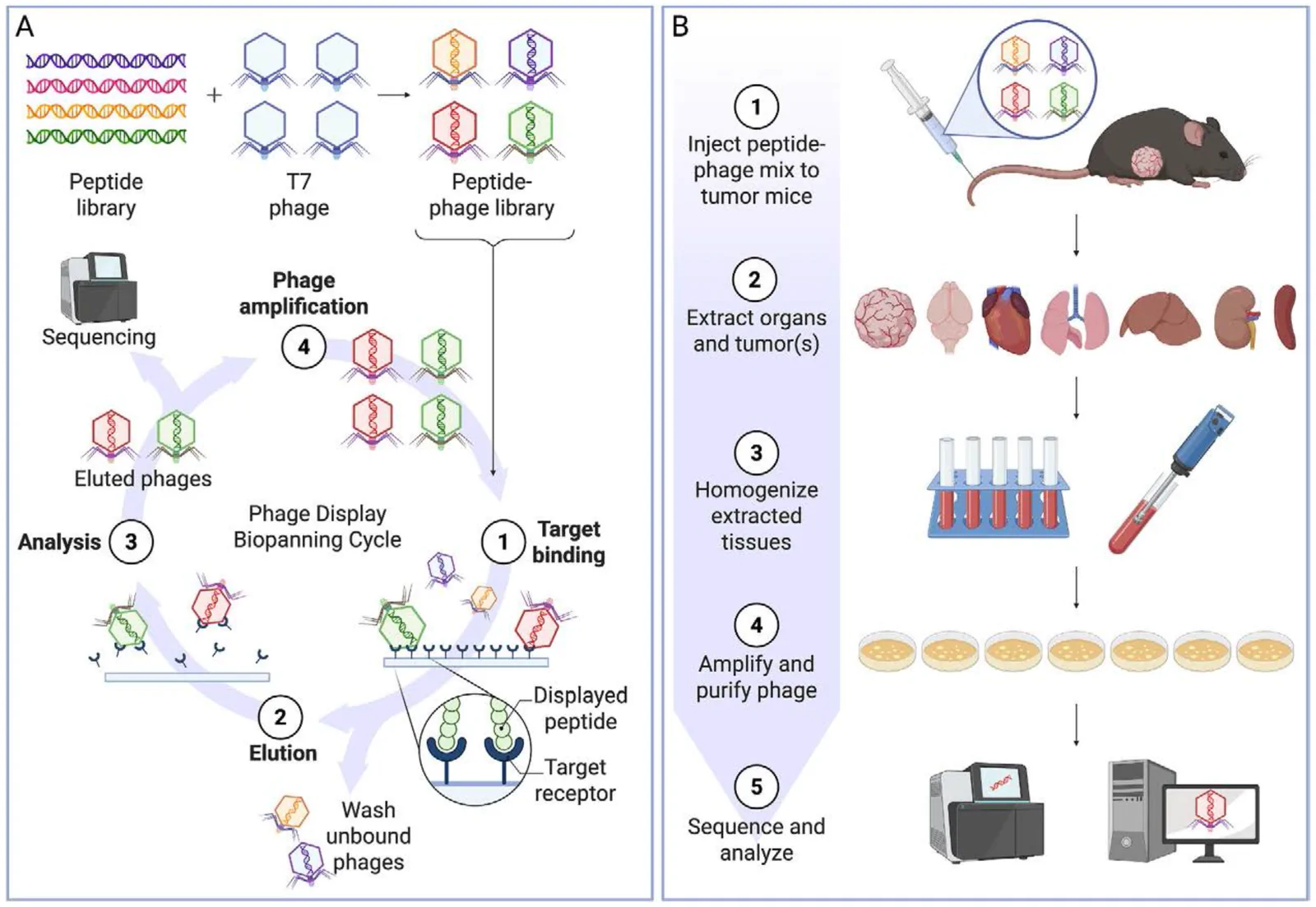
Schematic representation of *in vitro* (A) and *in vivo* (B) phage biopanning technology (adopted from [106]).

**Fig. 3.**
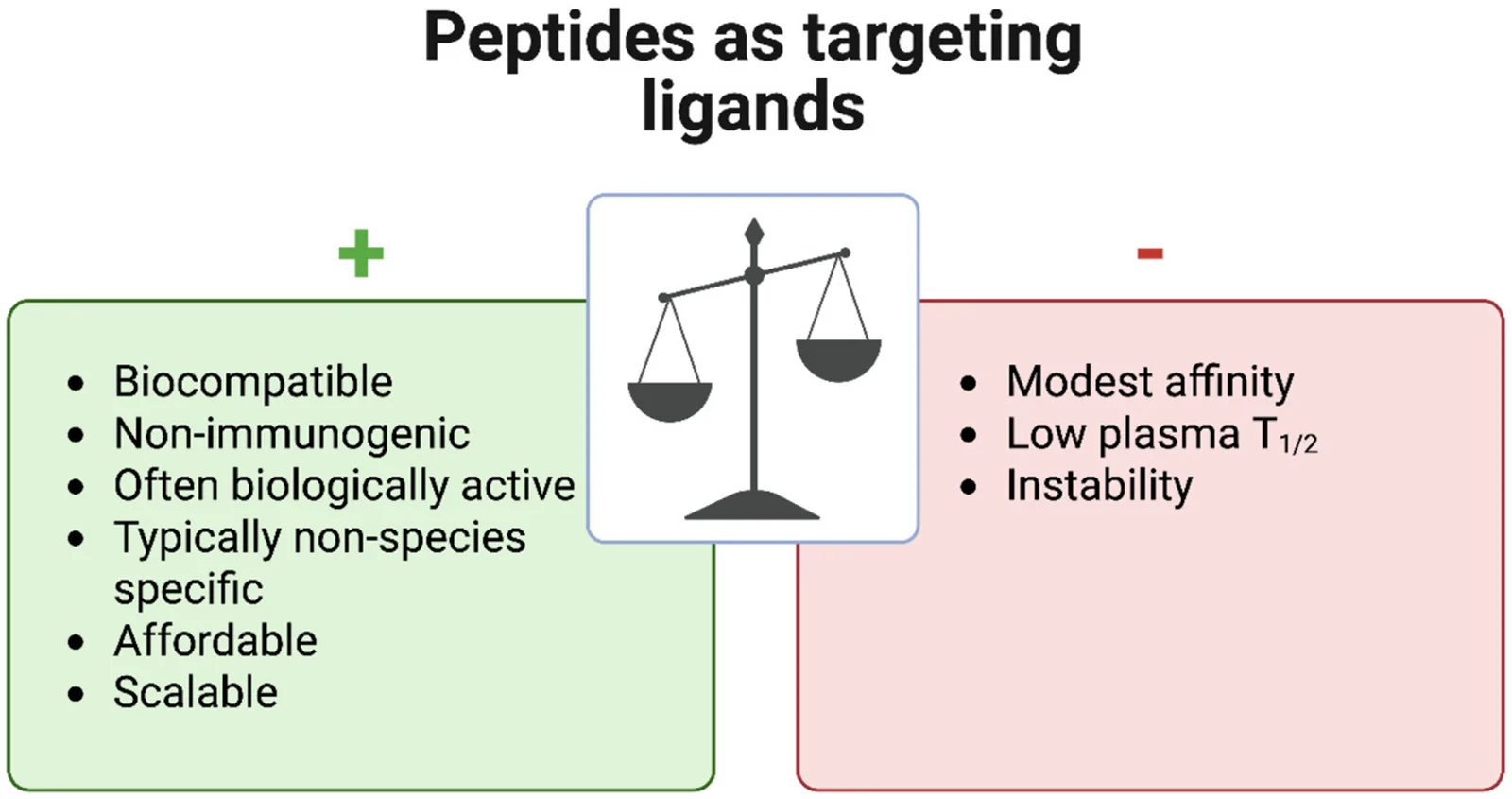
Advantages and limitations of vascular homing peptides as targeting ligands. Peptides offer several favorable properties, including biocompatibility, low immunogenicity, intrinsic biological activity, cross-species applicability, and cost-effective synthesis. However, they also have inherent challenges, such as relatively low binding affinity, short plasma half-life, and limited stability.

**Fig. 4.**
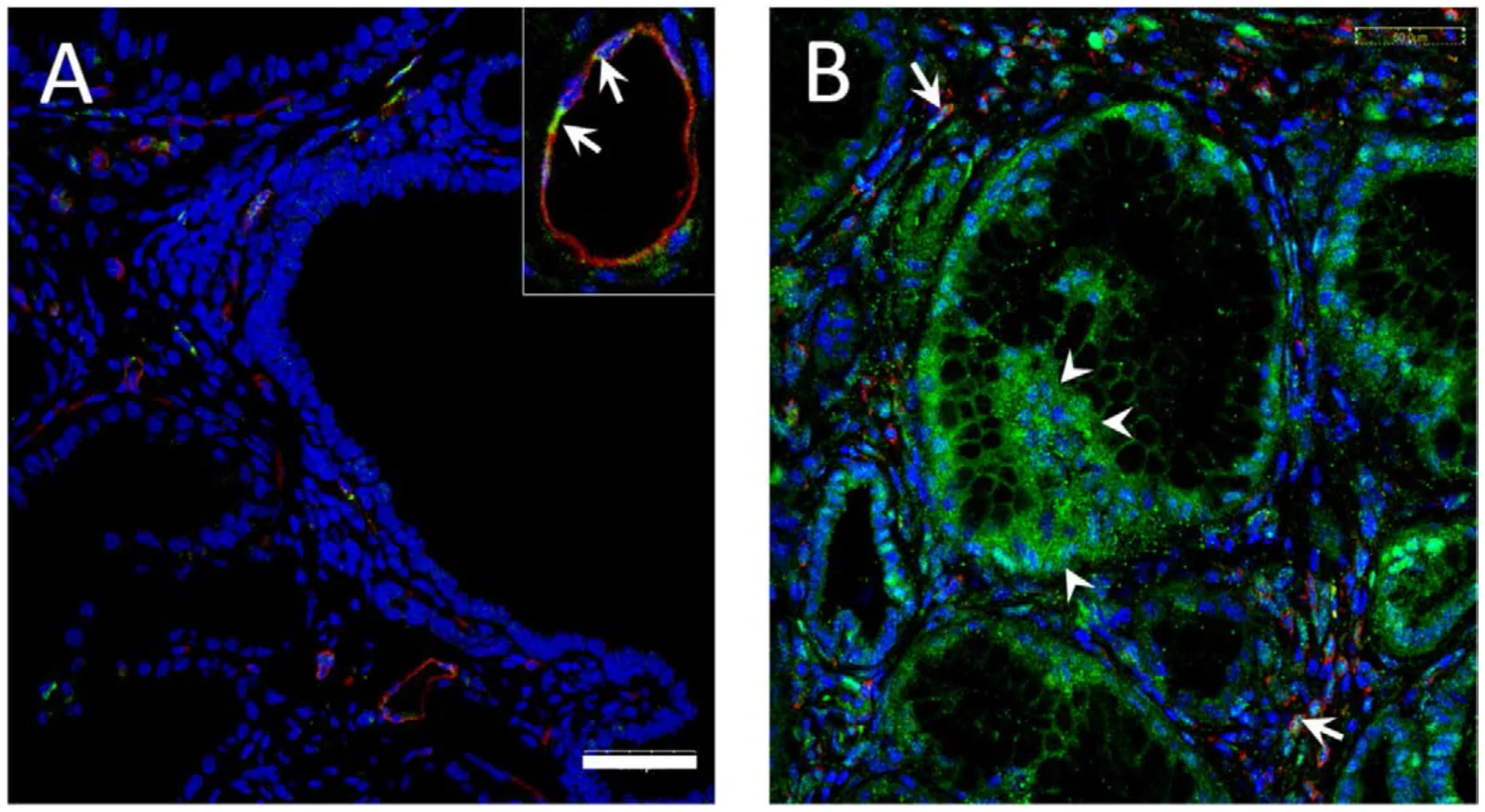
Vascular docking peptides vs. tumor-penetrating peptides (adopted from [91]). Mice bearing pancreatic ductal adenocarcinoma (PDAC) were intravenously injected with phages displaying the vascular docking CRGDC peptide (A) or the tumor-penetrating iRGD peptide (B). Tissue sections were stained with anti-T7 phage antibody (green), anti-CD31 antibody to visualize blood vessels (red), and DAPI nuclear stain (blue). The vascular docking peptide (A) shows phage binding primarily confined to the blood vessels (arrows), with minimal extravasation. In addition to vascular signal, the tumor-penetrating peptide iRGD (B) shows strong extravascular phage signal across the tumor parenchyma, including within the malignant ductal cells (arrowheads), demonstrating enhanced penetration after extravasation. Scale bar: 50 μm.

**Fig. 5.**
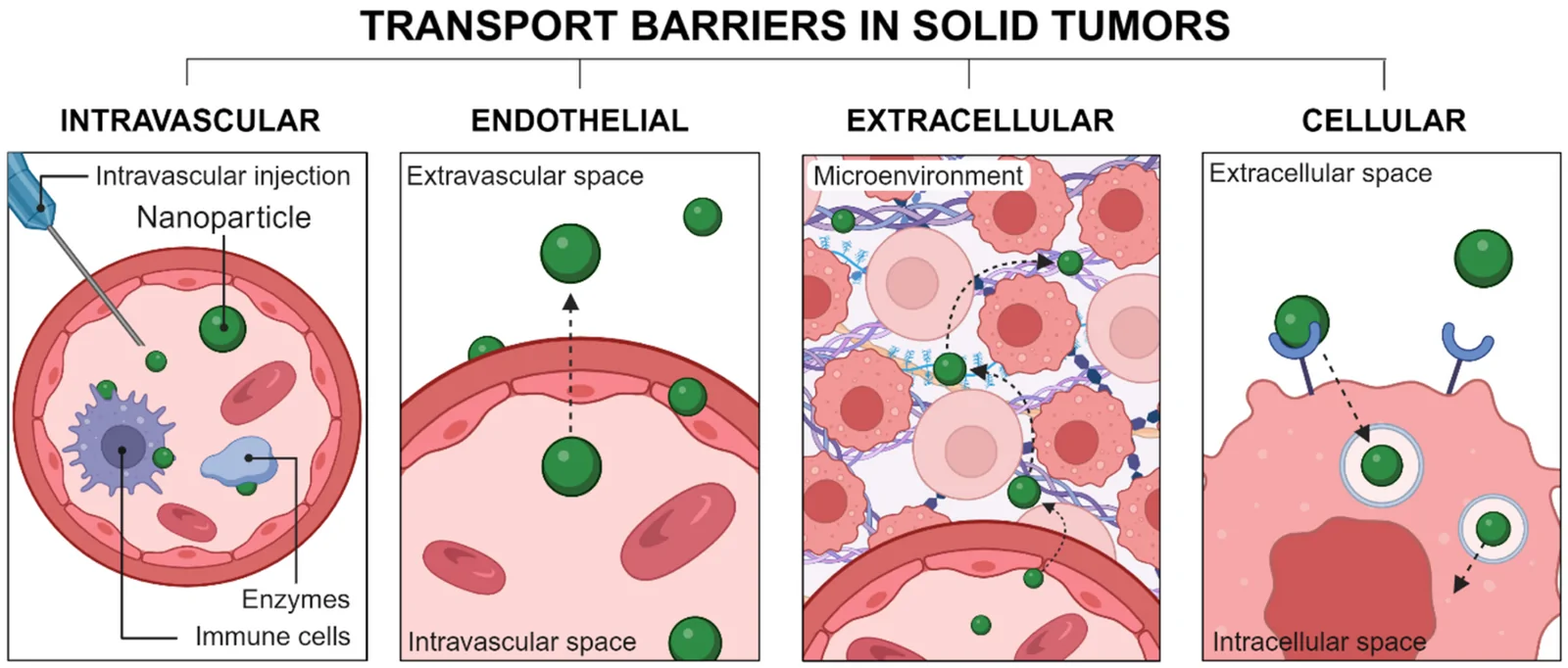
Biological barriers that NPs need to cross upon intravenous administration. These include intravascular (e.g., mononuclear phagocyte system, enzymes), endothelial (e.g., the endothelial cell layer, blood-brain barrier), extravascular (e.g., dense matrix, high interstitial pressure) and cellular (e.g., cell membrane, endosomal escape, efflux pumps) barriers. These complex and dynamic barriers must be navigated for successful NP-based drug delivery, and their interplay significantly impacts the efficacy of NP therapeutics [106].

**Fig. 6.**
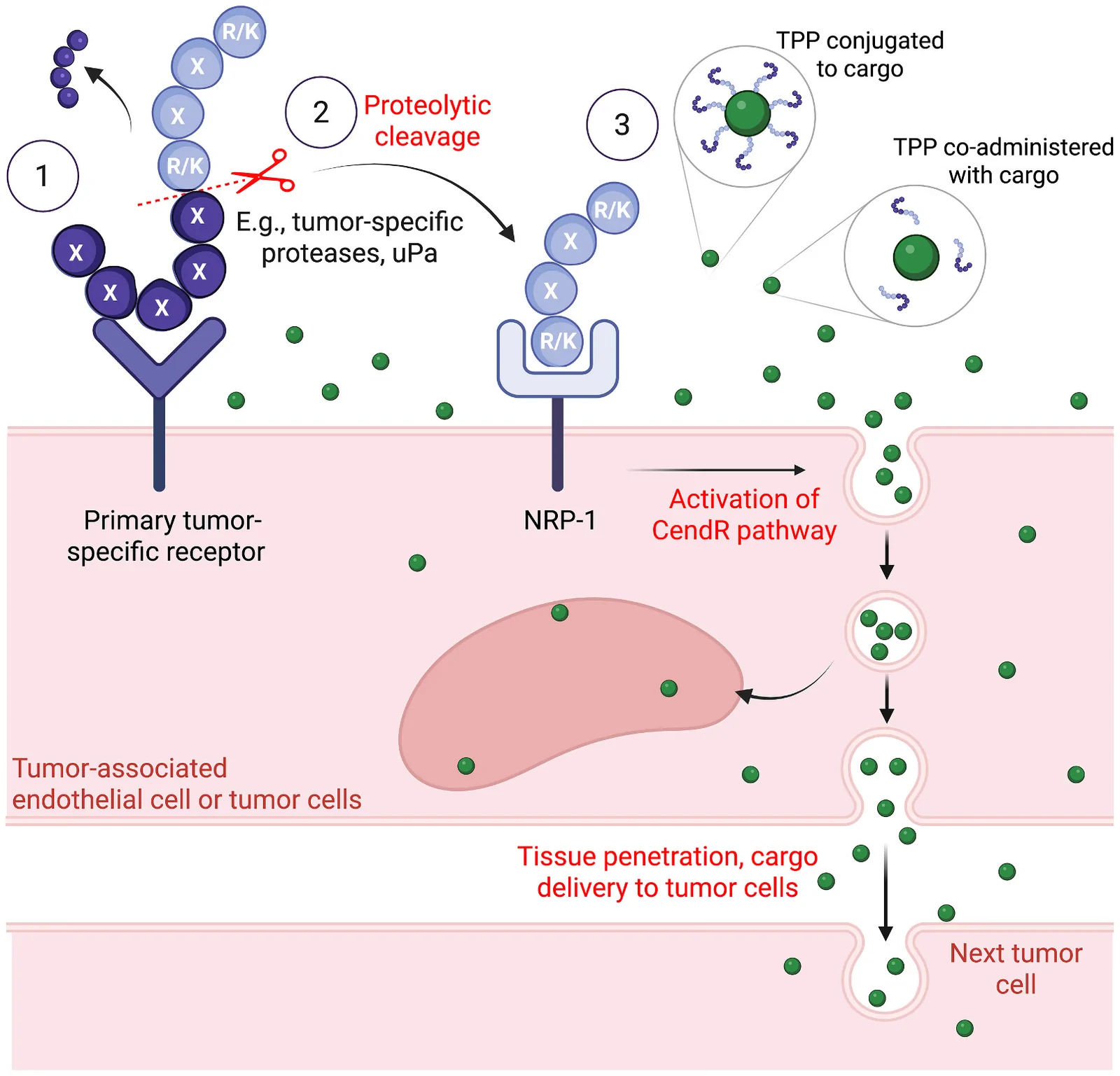
Tumor-penetrating peptides promote deep tissue access through a defined three-step mechanism. (1) the peptide binds to a primary receptor overexpressed on tumor cells or tumor-associated endothelial cells. (2) tumor-associated proteases cleave the peptide to expose a cryptic C-terminal CendR motif (R/KXXR/K). (3) The exposed motif binds to neuropilin-1 (NRP-1), triggering increased vascular and tissue permeability. This CendR-mediated pathway facilitates enhanced delivery of both co-administered compounds and peptide-conjugated cargos [91,154,162]. Adapted from [165] and created with BioRender (2025). https://BioRender.com/z7and9v.

## Data Availability

No data was used for the research described in the article.

## Appendix A. Supplementary data

Supplementary data to this article can be found online at https://doi.org/10.1016/j.jconrel.2025.114195.
