# Supplementary material for: Peptide-targeted nanoparticles for tumor therapy
Source: J Control Release. Author manuscript; Available in PMC 2026 Oct 26. (PMC13702853; doi:10.1016/j.jconrel.2025.114195)
Supplement: Supplementary file 1 [file NIHMS2214897-supplement-Supplementary_Materials_file.pdf]

Target	Peptide	NP system	Drug/active/imaging agent	Indication	Ref.
Integrins	Cilengitide	Coadministration	Verapamil, gemcitabine	Lung cancer (LLC, A549)	https://www.sciencedirect.com/science/article/pii/S153561081400419X?via%3Dihub
		Core–shell based nanocarrier (polymeric NPs)	Tumor necrosis factor (TNF)-related apoptosis inducing ligand (TRAIL)	Breast cancer (MDA-MB-231)	https://pubs.acs.org/doi/10.1021/acs.nanolett.5b04343
		Liposome	Superparamagnetic iron oxide nanoparticles (NPs; SPIONs) and quantum dots (QDs), MRI contrast agent	Rat glioma (tumor implantation)	https://onlinelibrary.wiley.com/doi/10.1002/adhm.201701130
		Microbubbles (gelatin and Poloxamer 188-grafted heparin copolymer)	Ultrasound-mediated microbubble destruction + MRI imaging	Rat Glioblastoma (C6)	https://www.sciencedirect.com/science/article/pii/S0168365916300116?via%3Dihub
		Chelator/peptide conjugate	NOPO chelator, 68Ga for PET/CT	Melanoma (M21 and M21L)	https://pubs.acs.org/doi/10.1021/mp5000746
		Thermosensitive liposomes	DOX	Pancreatic cancer (BxPC3)	https://onlinelibrary.wiley.com/doi/10.1002/advs.201902746
		Liposomes	Pep-1 peptide enhances anticancer efficacy of cilengitide, cilengitide here as a therapeutic agent	Glioma (U87)	https://www.sciencedirect.com/science/article/pii/S0927776517302138?via%3Dihub
		Coadministration with MRI imaging agent	MRI tracer (P1227)	Colorectal cancer (HT29)	https://link.springer.com/article/10.1245/s10434-013-3444-1
	SFITGv6	Labeled peptide	Peptides were labeled with radioactive isotopes such as 68Ga and 177Lu for imaging and potential endoradiotherapy	Oral cancer (HNO97), tumor-bearing patients	https://aacrjournals.org/clincancerres/article/23/15/4170/257598/Identification-of-a-Novel-ITG-v-6-Binding-Peptide
		Peptide (a modified version of a peptide with better binding capabilities – SFLAP3)	Peptides were labeled with radioactive isotopes such as 68Ga and 177Lu for PET imaging	Head and neck squamous cell carcinoma (HNO97, HNO399, and HNO223), tumor-bearing patients	https://jnm.snmjournals.org/content/59/11/1679
		Labeled peptide	Labeled with various isotopes such as Iodine-125 ([125I]), Lutetium-177 ([177Lu]), and Gallium-68 ([68Ga]) for PET/CT	Lung cancer (NCI-H2009 and NCI-H322), tumor-bearing patients	https://link.springer.com/article/10.1007/s11307-018-1296-6
	iRGD	Exosomes	Doxorubicin (DOX)	Breast cancer (MDA-MB-231)	https://www.sciencedirect.com/science/article/pii/S014296121301449X?via%3Dihub

		PLGA/lipid NPs	Photosensitizer (indocyanine green, ICG) and a hypoxia-activated prodrug (tirapazamine, TPZ)	Breast cancer (4T1)	https://pubs.acs.org/doi/10.1021/acsnano.6b08731
		Lipid-PEG NPs (ROS-responsive polyprodrug NPs)	Near-infrared (NIR)-emitting anticancer drug mitoxantrone (MTO)	Prostate cancer (LNCaP)	https://onlinelibrary.wiley.com/doi/10.1002/adma.201700141
		pH-sensitive polymeric NPs	siRNA	Prostate cancer (PC3)	https://onlinelibrary.wiley.com/doi/10.1002/anie.201601273
		Liposomes	Indocyanine green (ICG)	Breast cancer (4T1)	https://www.sciencedirect.com/science/article/pii/S0168365915303011?via%3Dihub
		Red-blood-cell (RBC)-mimetic NPs (PTX-loaded polymeric core and a hydrophilic RBC vesicle shell)	Paclitaxel (PTX)	Breast cancer (4T1)	https://onlinelibrary.wiley.com/doi/10.1002/adfm.201504780
		Mesoporous silica NPs (MSNPs)/silicasomes*	Irinotecan	Pancreatic cancer (KPC-derived PDAC)	https://www.jci.org/articles/view/92284
		Iron-based NPs (ultrasmall single-crystal Fe NPs (bcc-USINPs)	Fe (inducing ferroptosis and immunogenic cell death) in combination with PD-L1 immunotherapy	Hepatic and colorectal cancer (HepG2; MC38)	https://pubs.acs.org/doi/10.1021/jacs.1c07471
		Synthetic protein NPs based on polymerized human serum albumin (HSA)	siRNA against STAT3 (Signal Transducer and Activation of Transcription 3) (STAT3i)	Glioma (GL26)	https://www.nature.com/articles/s41467-020-19225-7
		Graphdiyne GDY–CeO2 nanocomposites (metallic)	miR181a as radiosensitizer (radiotherapy)	Esophageal cancer (tumor tissues embedded into mice)	https://onlinelibrary.wiley.com/doi/10.1002/adma.202100556
p32	Lyp-1/tLyp-1	Ultrasmall semimetal NPs of bismuth (Bi-LyP-1 NPs), metallic NP (LyP-1)	Bi for tomography/photoacoustic imaging and photothermal/radiotherapy (computed tomography/photoacoustic imaging and efficient synergistic NIR-II photothermal/radiotherapy of tumors.)	Breast cancer (4T1)	https://pubs.acs.org/doi/10.1021/acsnano.7b00476
		Liposomes (tLyp-1)	Vascular endothelial growth factor (VEGF) siRNA and chemotherapeutic docetaxel (DTX)	Brain tumor (U87 MG)	https://www.sciencedirect.com/science/article/pii/S014296121400252X?via%3Dihub
		Micelles (LyP-1)	Docetaxel (DTX); (NIR) dye-IR820 for photothermal/dynamic therapy (PDT/PTT)	Breast cancer (4T1)	https://www.sciencedirect.com/science/article/pii/S0142961216304021?via%3Dihub
		Mesoporous silica NPs (MSN) + Qdots (tLyP-1)	DOX, Qdots for near-infrared (NIR)-light-triggered photothermal therapy (PTT)	Breast cancer (4T1)	https://pubs.acs.org/doi/10.1021/acsnano.7b03088

		MMP-2 sensitive peptide-polymeric NP (IR820-CF2) (LyP-1)	DTX; IR820 for photothermal therapy; CF27 peptide =d-peptide antagonist for PD-1/PD-L1 blockage + Lyp-1	Breast, (4T1) and melanoma (MDA-MB-231, B16F10) cancers	https://onlinelibrary.wiley.com/doi/10.1002/adfm.201900004
		Phase-transformation NPs (lipid) (phase-transformation to microbubbles via acoustic droplet vaporization (ADV)) (tLyP-1)	Hydroxycamptothecin (10-HCPT low intensity focused ultrasound (LIFU)-assisted tumor ultrasound contrast agent	Breast cancer (MDA-MB-231)	https://pubs.acs.org/doi/10.1021/acs.nanolett.7b05087
		Gold and platinum NPs (metallic NPs) (LyP-1)	D-peptide antagonist of PD-L1, a MMP2-responsive peptide (PTT) (photothermal immunotherapy)	Breast cancer (4T1)	https://www.sciencedirect.com/science/article/pii/S0168365919303645?via%3Dihub
		Peptide-polymer-HA combination (tLyP-1)	DTX, D-a-tocopheryl succinate (TOS)	Prostate cancer (PC-3)	https://www.sciencedirect.com/science/article/pii/S0142961215007012?via%3Dihub
		PEG-PLA polymeric NPs, coadministration (tLyP-1)	PTX	Glioma (C6)	https://pubs.acs.org/doi/10.1021/mp400189j
		Heparin-quercetin conjugate (polymeric) (LyP-1)	Gambogic acid (GA), a chemotherapeutic agent, and quercetin (Qu), helps reverse MDR	Breast cancer (MCF-7)	https://www.sciencedirect.com/science/article/pii/S1742706118303295?via%3Dihub
	TT1/linTT1	Iron oxide NPs (nanoworms; NWs), micelles (LinTT1)	Pro-apoptotic/cytotoxic peptide [$_{\text{D}}$(KLAKLAK)$_2$]	Breast cancer (MCF10CA1a; 4T1; MMTV-PyMT)	https://pubs.acs.org/doi/10.1021/acs.nanolett.6b03815
		Iron oxide NWs, silver NPs (TT1)	Peptide was used for identification of compound #4014008, which binds specifically to the p32 protein.	Breast cancer (MCF10CA1a)	https://chemistry-europe.onlinelibrary.wiley.com/doi/10.1002/cbic.201500564
		Iron oxide NWs, silver NPs (AgNPs), albumin-PTX NPs (Abraxane) (LinTT1)	PTX, proapoptotic peptide [$_{\text{D}}$(KLAKLAK)$_2$]	Glioma (U87-MG, 005, WT-GBM, VEGF-KO-GBM, NCH421k)	https://www.sciencedirect.com/science/article/pii/S0168365919303281?via%3Dihub
		Iron oxide NWs (LinTT1)	Pro-apoptotic/cytotoxic peptide [$_{\text{D}}$(KLAKLAK)$_2$]; MRI imaging	Gastric (MKN-45P), ovarian (SKOV-3), and colon (CT-26) cancers	https://www.sciencedirect.com/science/article/pii/S0168365917306454?via%3Dihub
		MMP-sensitive PLA-based NPs (LinTT1)	PTX, DiR for imaging	Lung cancer (A549)	https://pubs.acs.org/doi/10.1021/acsami.7b11614
		Polymeric NPs (TT1), cofunctionalized with ACUPA (targets PSMA)	DOX and lapatinib (LAP)	Breast cancer (231Br)	https://www.sciencedirect.com/science/article/pii/S0168365920306015?via%3Dihub

		Polymersomes (polymeric NPs) (LinTT1)	Radioactive agent ^{124}I for PET imaging	Breast cancer (4T1)	https://www.oncotarget.com/article/24588/text/
		Polymersomes (LinTT1)	Novel anthracycline utorubicin and NiR for imaging	Breast cancer (MCF10CA1a)	https://onlinelibrary.wiley.com/doi/10.1002/anie.202016421
		Liposomes (Lipid NPs) (LinTT1)	Cytochrome C (Cyt C) and Lonidamine (LND)	Breast cancer (4T1)	https://onlinelibrary.wiley.com/doi/10.1002/adfm.202305316
		Protein nanocages (LinTT1)	MeTTMN, a near-infrared emissive AIEgen, which enhances fluorescence and ROS production for photodynamic therapy.	Hepatic cancer (HepG2)	https://www.sciencedirect.com/science/article/pii/S1742706123003859?via%3Dihub
Aminopeptidase N	NGR	Polymeric NPs	Near-infrared fluorophores DY-676 (for peptide-modified polymers) and DY-750	Colon, pancreatic cancer (CT26, BxPC3)	https://pubs.acs.org/doi/10.1021/nl404391r
		Graphene oxide (GO) sheets with silver (Ag) NPs deposited on them	DOX, PTT (Ag), X-ray contrast agent	Sarcoma (S180)	https://www.sciencedirect.com/science/article/pii/S0142961214003020?via%3Dihub
		Red blood cell membrane-coated solid lipid NPs	Vincristine	Brain cancer (C6)	https://pubs.acs.org/doi/10.1021/acsami.8b18664
		Thermosensitive liposomes	Cell penetrating peptide and DOX (CPP-DOX conjugate)	Fibrosarcoma (epithelial cancer, HT-1080)	https://www.sciencedirect.com/science/article/pii/S0142961214001203?via%3Dihub
		Peptide–lanthanide nanoclusters (peptide-metallic nanoclusters)	PMI (an MDM2-p53 interaction inhibitor), BIM (a pro-apoptotic peptide)	Colon cancer (HCT116)	https://pubs.acs.org/doi/10.1021/acsnano.8b00081
		Fullerene (C60)-based NPs (carbon-based), ROS sensitive linker	DOX; c60 is photosensitizer for PDT	Breast cancer (4T1)	https://www.sciencedirect.com/science/article/pii/S0168365916303753?via%3Dihub
		Liposomes	siRNA (c-myc); photolabile-caged cell-penetrating peptide (pcCPP) (NiR-triggered release)	Fibrosarcoma (epithelial cancer, HT-1080)	https://www.sciencedirect.com/science/article/pii/S0142961215000472?via%3Dihub
		Quantum dots (QDs)	Fluorescence imaging, MRI	Rat brain glioma model (C6)	https://www.sciencedirect.com/science/article/pii/S1549963416301460?via%3Dihub
		PEG-PLGA NPs	PTX; DiR for imaging	Glioma (U87)	https://www.sciencedirect.com/science/article/pii/S0142961214001264?via%3Dihub

		Metallic NPs	NIR and CT contrast agent	Hepatic cancer (HepG2)	https://pubs.rsc.org/en/content/articlelanding/2015/nr/c5nr05623c
Nucleolin	F3	Intrinsically radioactive UiO-66 nMOFs (nanoscale metal-organic frameworks)	DOX; positron-emitting isotope zirconium-89 (^{89}Zr) PET	Breast cancer (MDA-MB-231)	https://pubs.acs.org/doi/10.1021/acsnano.7b01530
		Liposomes	DOX and C6-ceramide (C6-Cer)	Breast cancer (MDA-MB-231)	https://www.sciencedirect.com/science/article/pii/S0142961215006596?via%3Dihub
		Polymeric NPs (PLA-PEG-PLA)	Photosensitizer for PDT (pyropheophorbide-a, PPa), PTX	Colorectal cancer (HCT-15)	https://pubs.acs.org/doi/10.1021/acsami.6b04442
		Polymeric micelles n(PEG-PLA)	PTX	Breast cancer (MCF-7)	https://pubs.rsc.org/en/content/articlelanding/2020/bm/c9bm02036e
		Peptide-based NIR-II probe	NIR-II fluorescent probe CH1055-F3, image-guided surgery	Breast cancer (4T1) and (DMBA)-induced spontaneous breast tumor rats	https://www.sciencedirect.com/science/article/pii/S1001841720302485?via%3Dihub
		PAMAM micelles (dendrimer core) polymeric NPs (pH-sensitive)	DOX and ^{64}CU for PET imaging	Breast cancer (MDA-MB-231)	https://www.sciencedirect.com/science/article/pii/S1742706118305038?via%3Dihub
		PLA-based nano-platform (polymeric NPs) (CREKA + F3 peptide conjugate added to NPs), pH-sensitive	EGFR-inhibitor erlotinib (EB); gamma secretase inhibitor (GSI)-DAPT	Breast cancer (MDA-MB-231)	https://www.tandfonline.com/doi/full/10.1080/10717544.2019.1576801
		Polypyrrole (PPy) NPs (polymeric)	DOX, PPy NIR absorbing PTT (thermal imaging-guided synergistic photothermal and chemical therapy)	Melanoma (C8161)	https://www.thno.org/v08p2634.htm
		PLGA (poly(lactic-co-glycolic acid)) NPs (polymeric)	Methylene blue (MB) - a sonosensitizer for sonodynamic therapy, and gadodiamide (Gd-DTPA-BMA), an MRI contrast agent	Breast cancer (MDA-MB-231)	https://www.dovepress.com/cell-penetrating-peptide-modified-nanoparticles-for-tumor-targeted-ima-peer-reviewed-fulltext-article-IJN
		Microbubbles (MBs) lipidic NPs	Ultrasound contrast agents	Syngeneic breast cancer tumors by transplanting a 1 mm3 piece of donor neu exon deletion line (NDL) tumor	https://www.sciencedirect.com/science/article/pii/S0142961216306445?via%3Dihub
Fibrin/fibronectin	CLT1	PEG-PLA NPs (polymeric)	PTX	Glioma (C6)	https://www.sciencedirect.com/science/article

					/pii/S0142961214000623?via%3Dihub
		Magnetic-fluorescent gadolinium(III)-doped carbon dots (Gd-CDs)	Gd MRI contrast agent, dually targeted with AS1411 aptamer	Renal cancer (RCC, 786-O)	https://www.sciencedirect.com/science/article/pii/S1385894722013018?via%3Dihub
		Generation 2 nanoglobulars (polylysine dendrimer with a silsesquioxane core)	Gd MRI and fluorescence imaging	Prostate cancer (PC3-GFP)	https://pubmed.ncbi.nlm.nih.gov/23471641/
		Nanoscavengers (ECMT NS) consisting of NPs	Digestive enzyme chymotrypsin, catalase, calcium peroxide, photosensitizer chlorin e6, (cocktail calcium ion/photodynamic/immune therapy)	Melanoma (B16F10)	https://www.sciencedirect.com/science/article/pii/S136970212200270X?via%3Dihub
		Microbubbles (lipids)	PTX, ultrasound therapy, cotargeted with cRGD	Pancreatic cancer (PANC-1)	https://pubs.acs.org/doi/10.1021/acsbiomaterials.2c00069
	ZD2	Gold nanostars (metallic NPs)	Photothermal therapy (PTT) + MRI	Breast cancer (MDA-MB-231)	https://onlinelibrary.wiley.com/doi/10.1002/adhm.201801144
		Fluorescently labeled peptide	Cy5 for imaging	Prostate cancer (PC3)	https://pubs.acs.org/doi/10.1021/acs.bioconjchem.5b00178
		Gadofullerenes, specifically tri-gadolinium nitride metallofullerene (Gd3N@C80) (metallic NPs)	Gd(III) ions for MRI	Breast cancer (MDA-MB-231, MCF-7)	https://www.nature.com/articles/s41467-017-00741-y
		Peptide-contrast agent	Gd for MRI, Cy5.5 for imaging	Prostate cancer (PC3)	https://pubs.acs.org/doi/10.1021/acs.bioconjchem.6b00719
		Targeted MRI contrast agent (-Gd(HP-DO3A) (MT218)	Gd for MRI, (used to assess the tumor response and therapeutic efficacy of the RGD-PEG-ECO/miR-200c)	Breast cancer (MDA-MB-231, Hs578T TNBC)	https://link.springer.com/article/10.1007/s11095-021-03083-z
		Dextran-peptide conjugates	Dextran for MRI imaging	Pancreatic cancer (KPC-derived)	https://pubs.acs.org/doi/10.1021/acs.bioconjchem.9b00161
		Peptide-targeted MRI contrast agent, ZD2-Gd(HP-DO3A)	Gd for MRI	Breast cancer (4T1 and MDA-MB-231)	https://onlinelibrary.wiley.com/doi/10.1002/mrm.26976
		Peptide Gd–DOTA conjugates	Gd for MRI	Prostate cancer (PC3)	https://pubs.acs.org/doi/10.1021/acs.molpharmaceut.7b00619
		Targeted MRI contrast agents based on Gd(HP-DO3A)	Gd for MRI	Prostate cancer (PC3, LNCaP)	https://pubs.acs.org/doi/10.1021/acsmedchemlett.8b00172

		ZD2-N3-Gd (HP-DO3A) (MT218), a targeted MRI contrast agent	Gd for MRI	Pancreatic cancer (Capan-1, BxPC3, PANC-1)	https://www.frontiersin.org/journals/oncology/articles/10.3389/fonc.2020.586727/full
		SPECT probe 99mTc-HYNIC-ZD2	99mTc for SPECT	Breast cancer (MDA-MB-468)	https://pubs.acs.org/doi/10.1021/acsomega.7b00226
	CREKA	Peptide-Gd coMRI contrast agent	Gd for MRI	Breast tumor (4T1)	https://www.nature.com/articles/ncomms8984
		Iron oxide NPs	MRI and MPI contrast agent, magnetic hyperthermia therapy	Breast tumor (4T1)	https://pubs.acs.org/doi/10.1021/acs.nanolett.9b00630
		Micelles	Cy7-labeled for imaging	Glioma (GL261)	https://www.sciencedirect.com/science/article/pii/S0142961213013082?via%3Dihub
		Multi-walled carbon nanotubes	Photothermal therapy, NIR	Lung cancer (A549)	https://www.sciencedirect.com/science/article/pii/S0142961215009710?via%3Dihub
		Polyamidoamine (PAMAM) dendrimers	FITC and IR-783 for imaging	Glioblastoma (U87MG)	https://www.sciencedirect.com/science/article/pii/S0021979715002787?via%3Dihub
		Ultrasmall manganese ferrite NPs	MRI detection	Breast tumor (4T1)	https://onlinelibrary.wiley.com/doi/10.1002/adma.201906799
		Polymer NPs targeting CAFs + triptolide-loaded micelles	TCM α-mangostin (interfering of TGF-β/Smad signaling pathway) for vascular normalization in tumors, after pre-treatment triptolite-loaded micelles kill tumor	Pancreatic tumor (PANC-1, NIH3T3)	https://www.sciencedirect.com/science/article/pii/S0142961220301538?via%3Dihub
		Liposomes	Platelet inhibitor ticagrelor	Breast tumor (4T1)	https://www.thno.org/v07p1062.htm
		Liposomes	DOX	Breast tumor (4T1)	https://www.sciencedirect.com/science/article/pii/S0168365917310969?via%3Dihub
		Enzyme sensitive gold NPs	Au NIR for PTT, NPs assembele into bigger composits in tumors (triggered by alkaline phosphatase) improved PTT	Prostate cancer (PC-3)	https://pubs.rsc.org/en/content/articlelanding/2018/cc/c8cc05136d

Tumor-associated macrophages (TAMs)	M2pep	Lipid NPs	Anti-colony stimulating factor-1 receptor (anti-CSF-1R) small interfering RNA (siRNA), cotargeted with α-peptide (a scavenger receptor B type 1 (SR-B1) targeting peptide)	Melanoma (B16F10)	https://pubs.acs.org/doi/10.1021/acsnano.7b05465
		Micelles	PI3K-γ inhibitor NVP-BEZ 235 and CSF-1R-siRNA, CpG -toll-like receptor 9 agonist	Pancreatic cancer (Pan 02)	https://www.sciencedirect.com/science/article/pii/S0168365920300870?via%3Dihub
		PLGA-NPs (polymeric NPs)	Baicalin and melanoma antigen Hgp peptide fragment 25-33, cotargeted with α-peptide (a scavenger receptor B type 1 (SR-B1) targeting peptide)	Melanoma implants (B16)	https://www.thno.org/v11p2892.htm
		Human ferritin heavy chain (rHF) nanocages (protein NPs)	CpG -toll-like receptor 9 agonist	Breast cancer (4T1)	https://pubs.rsc.org/en/content/articlelanding/2020/nr/d0nr04520a
		Poly(lactic-co-glycolic acid) NPs (polymeric)	PLX3397, an inhibitor of the colony stimulating factor-1 (CSF-1)/CSF-1 receptor (CSF-1R)	Melanoma (B16F10)	https://link.springer.com/article/10.1007/s11095-019-2596-5
		A poly(lactide-co-glycolide) NPs	D-lactate (DL; a gut microbiome metabolite) acts as an endogenous immunomodulatory agent that enhances Kupffer cells for clearance of pathogens	Hepatic cancer (Hepa1-6)	https://www.science.org/doi/10.1126/sciadv.adg2697
		Cyclic modification of M2pep with (K9R and W10Y) - M2pep(RY)) labeled with Cy5	Cy5 for imaging	Colon (CT-26) and breast (4T1)cancers	https://www.thno.org/v06p1403.htm
		Superparamagnetic Iron oxide NPs (SPION)	IO NPs for magnetic hyperthermia therapy + MRI imaging	Breast cancer (4T1)	https://www.sciencedirect.com/science/article/pii/S1005030221000190?via%3Dihub
		Liposomes	Zoledronic acid (ZA) in the core, loads hematoporphyrin monomethyl ether (HMME, a typical sonosensitizer), sonodynamic therapy + immunotherapy	Breast cancer (4T1)	https://www.sciencedirect.com/science/article/pii/S1742706123000764?via%3Dihub
		Er-based rare-earth NPs	Second near-infrared (NIR-II) fluorescence, BBB opening by microbubbles and FUS, MRI	Glioblastoma (U87MG)	https://onlinelibrary.wiley.com/doi/10.1002/smll.202202201
	UNO/mUNO	FAM-peptide, polymersomes (UNO)	FAM for imaging, PTX	Breast (4T1 and MCF-7) melanoma, (B16F10) melanoma, glioma (WT-GBM), gastric carcinoma (MKN45-P)	https://www.nature.com/articles/s41598-017-14709-x

		Lignin NPs (LNPs) (polymeric) (mUNO)	Dual agonist of the toll-like receptors TLR7/8 (resiquimod, R848), loaded LNPs coadministered with vinblastine (Vin)	Breast cancer (4T1)	https://www.sciencedirect.com/science/article/pii/S1742706120305614?via%3Dihub
		FAM-peptide polymersomes (mUNO)	FAM for imaging	Breast cancer (4T1)	https://pubs.acs.org/doi/10.1021/acs.molpharmaceut.0c00226
		Star-shaped polyglutamate (St-PGA) (peptide-polymer nanoconjugate) (mUNO)	DOX	Breast cancer (4T1)	https://aacrjournals.org/cancerrescommun/article/2/6/533/705241/Depletion-of-Mannose-Receptor-Positive-Tumor
		Evans Blue (EB)-Based NPs, EB binds with albumin forming NPs in vivo (mUNO)	Indocyanine green (ICG) NIR and generates ROS and PDT, Ferrocene to potentiate PDT, in combination with anti- PD-1 mAbs	Breast tumor (4T1)	https://www.frontiersin.org/journals/chemistry/articles/10.3389/fchem.2024.1469568/full
	RP-182	Biodegradable poly (β-amino ester)-based NPs	DNA encoding an ErbB2-specific chimeric antigen receptor (CAR) for in situ genetic editing of macrophages.	Glioma (GL261-H)	https://pubmed.ncbi.nlm.nih.gov/36805678/
		Peptide	Peptide to target and reprogram tumor-associated macrophages (TAMs).	Colon (CT-26) and melanoma (B16) cancer models	https://www.science.org/doi/10.1126/scitranslmed.aax6337
	CRV	pH/GSH-responsive polymer NPs camouflaged with CRV-expressing lung cancer cell membranes (CRV-LLCM)	dBET6, a BRD4-targeted PROTAC, to induce apoptosis in lung cancer cells and tumor-associated macrophages (TAMs).	Lung cancer (LLC)	https://onlinelibrary.wiley.com/doi/10.1002/advs.202202039
		Porous silicon NPs (pSiNPs)	Sulforhodamine 101 (SR101) for imaging	Breast cancer (4T1, MCF10CA1a), pancreatic cancer (KRAS-Ink), lung cancer (H1975, LL/2)	https://www.sciencedirect.com/science/article/pii/S0168365919301555?via%3Dihub
		Zwitterion-type NIR Aggregation-Induced Emission Luminogens (AIEgens) (BITT) coated in CRV-engineered exosome membranes (CRV-EM)	Aggregation-Induced Emission Luminogens (AIEgens) (BITT) for PTT/PDT	Lung cancer (LLC)	https://jnanobiotechnology.biomedcentral.com/articles/10.1186/s12951-023-01799-1
Fibronectin Extra Domain-B (FN-EDB) and Tenascin-C (TNC)	PL1/PL3	Iron oxide NPs NWs; silver NPs (PL1)	Pro-apoptotic/cytotoxic peptide [D(KLAKLAK)$_2$], IO NPs for MRI	Glioblastoma (NCH421K, U87MG and, WT-GBM), prostate carcinoma (PC3)	https://www.sciencedirect.com/science/article/pii/S0142961219304727?via%3Dihub
		Iron oxide NWs and metallic silver NPs (AgNPs) (PL3)	Pro-apoptotic/cytotoxic peptide [D(KLAKLAK)$_2$],	Glioblastoma (U87-MG, WT-GBM) and prostate carcinoma (PC3)	https://pubmed.ncbi.nlm.nih.gov/32242067/

		uPa-cleavable peptide derivative containing cryptic CendR element (PL3uCendR)	FAM-labeled peptide for imaging	Glioblastoma (WT-GBM, VEGF-KO-GBM, NCH421k, U87-MG)	https://pubmed.ncbi.nlm.nih.gov/39012578/
Hyaluronan	IP3	AgNPs	FAM for imaging	Peritoneal carcinomatosis of gastric origin (MKN-45P), colon cancer (CT26)	https://journals.sagepub.com/doi/10.1177/1010428317701628
Laminin/nidogen complex	CSG	Fusion protein, the TNFα-CSG, superparamagnetic iron-oxide NPs (IO-NP)	Tumor necrosis factor alpha (TNFα) to improve tumor perfusion, MRI	Spontaneous hepatic and pancreatic cancer	https://www.frontiersin.org/journals/oncology/articles/10.3389/fonc.2022.837234/full
		Superparamagnetic iron-oxide NPs (IO-NP), CREKA vs CSG targeted (CSG worked better)	Tumor necrosis factor alpha (TNFa) to improve tumor perfusion, MRI	Spontaneous pancreatic cancer (RIP1-Tag5)	https://pmc.ncbi.nlm.nih.gov/articles/PMC8539756/
		Iron oxide NPs (IONPs), fusion protein, the TNFa-CSG,	Gadoteridol as contrast agents, tumor necrosis factor alpha (TNFa) to improve tumor perfusion, MRI	Breast (4T1, MDA-MB-435), pancreatic (RIP1-Tag5, CT26), hepatic (ALB-Tag), cancers	https://pubmed.ncbi.nlm.nih.gov/31709774/
Epidermal growth factor receptor (EGFR)	EBP	Peptide–phthalocyanine conjugates	Zinc(II) phthalocyanine photosensitizers for photodynamic therapy (PDT)	Colorectal cancer (HT29)	https://pubs.acs.org/doi/10.1021/acs.jmedchem.0c01677
		Peptide-drug conjugate	DOX	Lung cancer (A549)	https://www.sciencedirect.com/science/article/pii/S0022354915000210?via%3Dihub
	GE11	Hyaluronic acid nanogels	Therapeutic proteins such as cytochrome C (CC) and Granzyme B (GrB)	Ovarian (SKOV-3) and breast (MDA-MB-231) cancers	https://pubs.acs.org/doi/10.1021/acsami.7b06879
		PLGA NPs (polymeric)	Curcumin, an anti-cancer agent	Breast cancer (MCF-7)	https://pubs.rsc.org/en/content/articlelanding/2017/nr/c7nr06898k
		Liposomes	DOX	Lung cancer (A549)	https://www.dovepress.com/ge11-modified-liposomes-for-non-small-cell-lung-cancer-targeting-prepa-peer-reviewed-fulltext-article-IJN
		MS2 virus-like particles (VLPs)	Long non-coding RNA (lncRNA) MEG3	Hepatic cancer (HepG2)	https://www.oncotarget.com/article/8115/text/
		Self-assembly amphiphilic peptide NPs	Gemcitabine and the PARP inhibitor Olaparib	Pancreatic cancer (capan-1)	https://pubs.acs.org/doi/10.1021/acsnano.8b01573
		Selenium NPs (metallic)	Oridonin	Esophageal cancer (KYSE-15)	https://www.tandfonline.com/doi/full/10.1080/1

					0717544.2017.1386729#abstract
		Unimolecular micelles NPs based on an aptamer	Aminoflavone (AF)	Breast cancer (MDA-MB-468)	https://www.sciencedirect.com/science/article/pii/S0142961216302265?via%3Dihub
		PLGA-based NPs (polymeric)	DTX and pH-sensitive curcumin (CUR) prodrug	Prostate cancer (LNCaP)	https://www.sciencedirect.com/science/article/pii/S0753332216323496?via%3Dihub
		GSH-sensitive polymersomes (Doxil as a ctrl)	DOX	Liver cancer (SMMC7721)	https://www.sciencedirect.com/science/article/pii/S1742706117306293?via%3Dihub
		Redox (GSH)-sensitive polymeric micelles	Gemcitabine (GEM), miR-519c - a microRNA that helps sensitize cancer cells to GEM	Pancreatic cancer (PaCa-2R, hPSCs)	https://www.science.org/doi/10.1126/sciadv.abd6764
		Boron Dipyrromethene (BODIPY) bioconjugates	To evaluate the tumor localization and imaging (NIR imaging) capabilities of the BODIPY conjugates. Glucose or biotin units were added to improve cellular uptake (glucose) and improve solubility (biotin)	Colon cancer (HT-29)	https://pubs.acs.org/doi/10.1021/acs.jmedchem.8b01746
	D4	Radiolabeled peptide conjugate	Radiolabeled D4 peptide conjugate with 99mTc for imaging	Lung cancer (A549)	https://www.tandfonline.com/doi/full/10.2217/fon-2016-0426
		Chemically self-assembled nanorings (CSANs) made from dihydrofolate reductase (DHFR) fusion proteins (PEGylated Protein Nanostructures)	Radiolabel DOTA[^{64}Cu] for microPET/CT imaging and gamma counting	Brain cancer (U87 MG)	https://pubs.acs.org/doi/10.1021/acs.molpharmaceut.6b00110

Supplementary Table 1. Summary of selected tumor-homing peptides and their applications in nanomedicine

Data presented in this table were compiled through a comprehensive literature review using the Scopus database. Search terms included tumor-penetrating and homing peptides (e.g., “cilengitide,” “iRGD,” “Lyp-1,” “tLyp-1,” “TT1,” “LinTT1,” “iNGR,” “NGR,” “F3,” “CLT1,” “CREKA,” “EBP,” “M2pep,” “uno,” “muno,” “PL1,” “PL3,” “IP3,” “RP-182,” “CRV,” “CSG,” “GE11,” “ZD2,” “DAG,” “sfitgv6,” and “D4”) combined with keywords related to delivery and cancer (e.g., “peptide,” “nanoparticle*,” “nanomedicine*,” “deliver*,” “homing,” “tumor,” “cancer,” “neoplasm,” and “malignant”). The search was limited to peer-reviewed articles published between 2014 and 2024, prioritizing the most highly cited research in each peptide category. These searches were supplemented by manual reviews of

reference lists in key articles, exploration of relevant journals, and consultation with experts in the field. The advanced Scopus queries incorporated filters to limit results to the top 10 cited articles per search term, ensuring the inclusion of high-impact studies within the defined timeframe. This methodology was designed to ensure a thorough and focused review of relevant literature for this study.
